# Efficient therapeutic delivery by a novel cell-permeant peptide derived from KDM4A protein for antitumor and antifibrosis

**DOI:** 10.18632/oncotarget.8682

**Published:** 2016-04-11

**Authors:** Hu Wang, Jie-Lan Ma, Ying-Gui Yang, Yang Song, Jiao Wu, Yan-Yan Qin, Xue-Li Zhao, Jun Wang, Li-Li Zou, Jiang-Feng Wu, Jun-Ming Li, Chang-Bai Liu

**Affiliations:** ^1^ The Institute of Cell Therapy, China Three Gorges University, Yichang 443002, China; ^2^ Medical School, China Three Gorges University, Yichang 443002, China; ^3^ Hubei Key Laboratory of Tumor Microenvironment and Immunotherapy, China Three Gorges University, Yichang 443002, China; ^4^ The 1st People's Hospital of Yichang, Yichang 443000, China

**Keywords:** cell-penetrating peptides (CPPs), internalization, GFP, melanoma, anti-tumor

## Abstract

Cell-penetrating peptide (CPP) based delivery have provided immense potential for the therapeutic applications, however, most of nonhuman originated CPPs carry the risk of possible cytotoxicity and immunogenicity, thus may restricting to be used. Here, we describe a novel human-derived CPP, denoted hPP10, and hPP10 has cell-penetrating properties evaluated by CellPPD web server, as well as *In-Vitro* and *In-Vivo* analysis. *In vitro* studies showed that hPP10-FITC was able to penetrate into various cells including primary cultured cells, likely through an endocytosis pathway. And functionalized macromolecules, such as green fluorescent protein (GFP), tumor-specific apoptosis inducer Apoptin as well as biological active enzyme GCLC (Glutamate-cysteine ligase, catalytic subunit) can be delivered by hPP10 *in vitro* and *in vivo*. Collectively, our results suggest that hPP10 provide a novel and versatile tool to deliver exogenous proteins or drugs for clinical applications as well as reprogrammed cell-based therapy.

## INTRODUCTION

Low cell membrane permeability of biologically active molecules that specifically label cellular structures or control biological functions has been a limitation difficult to overcome from basic research to therapeutics. Although electroporation, microinjection, lipid-based transfection, and viral vectors have already been widely used, they still have drawbacks such as inefficient, cytotoxic, complex, penurious bioavailability and unreliable for *in vivo* studies [1, 2]. Thus, many alternative approaches [3–5] especially peptide-based transduction for foreign bioactive molecule delivery have been discovered and exploited.

Cell-penetrating peptides (CPPs) also termed as PTDs (protein transduction domains) or membrane-translocating sequences, are small peptides contain 6 to 30 amino acid residues in length, these distinctive peptides have biomedical potential as noninvasive and minimally cytotoxic agents for membrane translocation via endocytosis and/or macropinocytosis [6–8]. Three different classes of CPPs (cationic, hydrophobic and amphipathic) were divided based on their biophysical properties. Among the identified CPPs, the first discovered HIV-1 TAT peptide (residues 48–60), MPG peptide (HIV-1 gp41 and SV40 large T antigen fusion peptide), VP22, Antp (Drosophila Antennapedia transcription factor), Pep1, R7 or R9 [7, 9, 10] have been widely used for peptide-based molecule delivery systems, including cellular delivery of therapeutic molecule cargos, which range from peptides, proteins, nucleic acids-based macromolecules (DNA, RNA and siRNA duplexes), and small chemical compounds as well as nano-sized particles [1, 11, 12]. Accordingly, CPP have demonstrated utility as valuable vehicle for intracellular delivery of macromolecules, cargo has to be covalently [13] or non-covalently [14] linked to CPPs for efficient uptake. In recent years, numerous applications for CPPs not only have been shown from a vast number of fundamental researches (including CPP-based somatic cell reprogramming [5, 15–18], CPP-mediated genome editing [5, 19–21]), but also a little number of CPP-coupled molecules has entered into a phase II clinical trials [6].

Although CPP-based proteins delivery have provided immense opportunities for the safest and most useful application in therapeutic application, the cytotoxicity and the transport efficiency of each CPP cargo complex must be carefully optimized. Previous studies have shown that the cytotoxicity and uptake efficiency of CPP-conjugate is cargo-dependent [22], while penetrating efficiency of different CPPs may also vary according to the peptide sequence, cell type and cell-membrane carbohydrate composition [7]. We have previously shown that small molecule DMSO (dimethyl sulfoxide) [23] and BIT (1, 2-benzisothiazolin-3-one) [24] can be used to facilitate the penetrating efficiency of TAT or TAT-Apoptin (Apoptin, VP3 protein from chicken anaemia virus) conjugates for a range of cell types. Furthermore, some successful attempts have been made to develop algorithm and/or computational methods for CPPs predicting [1, 25–28], non-penetrating and penetrating peptides can easily be screened from known protein sequences, although the exact mechanism for the cellular entry of CPPs is still debated [29–31]. A large number of studies have shown the possibility of CPPs as drug delivery tools for human disease treatment; however, most of their nonhuman originated CPPs carry the risk of possible cytotoxicity and immunogenicity, such as classical CPPs of HIV-1 TAT, thus restricting to be used as intracellular delivery of potential therapeutics. To overcome this limitation, the human-originated CPPs identification is quite valuable.

Here in this study, we have found a human-derived penetrating peptide (hPP10) from C-terminal fragment of KDM4A, and evaluated its penetrating potential for different cargos delivery. Various types of cell internalization, tumor tissue penetration and cytotoxicity of the fusion protein, as well as anti-hepatic fibrosis of hPP10 conjugation with functional enzyme were examined *in vitro* and *in vivo*. Our results strongly suggest that delivery of the biological active protein using hPP10 is suitable for both *in vitro* and *in vivo* useage, as well as for translation into clinical therapies.

## RESULTS

### Identification of potential human-derived CPPs in *in silico* approach

**Figure 1:**
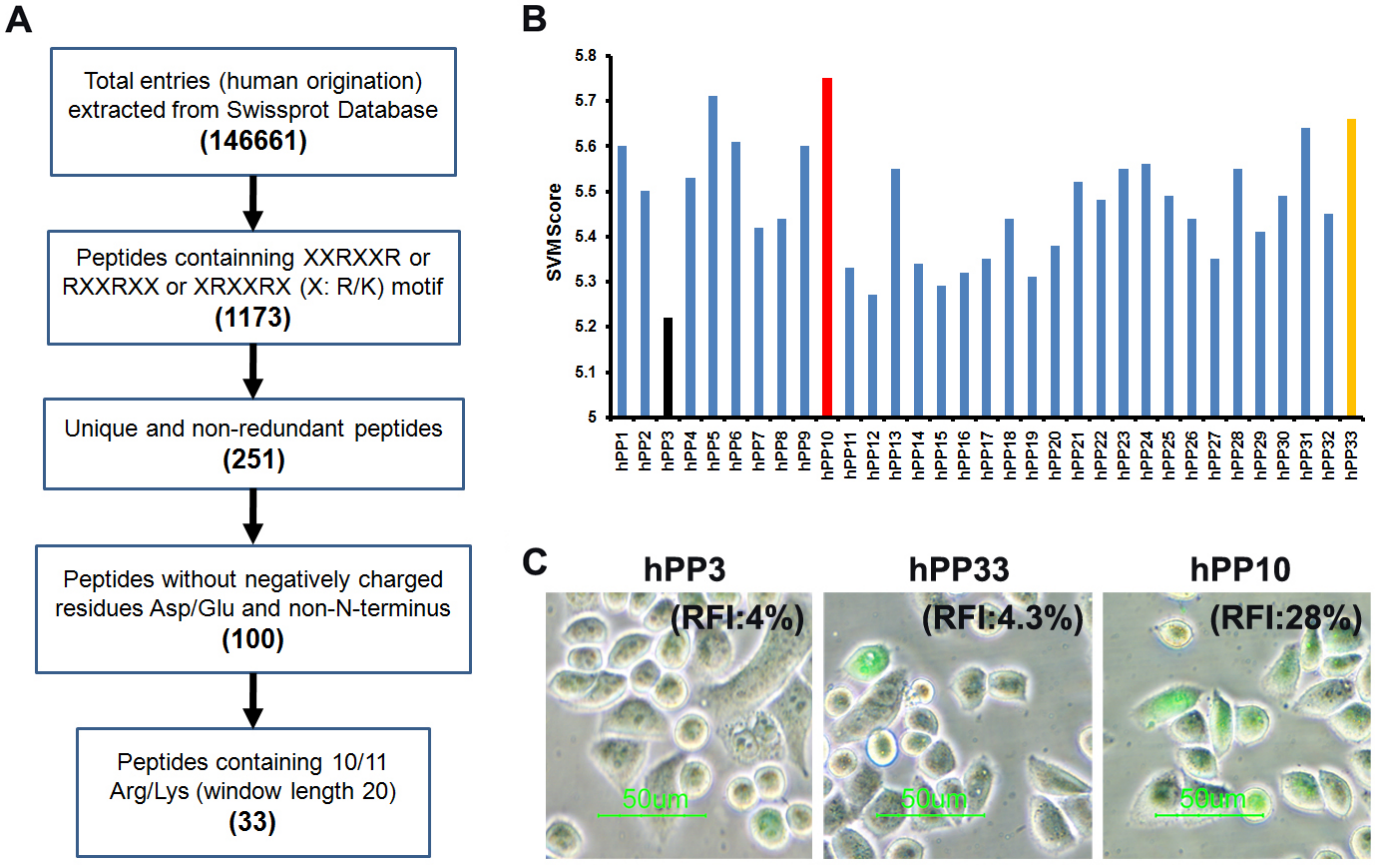
Identification of human-derived cell-penetrating peptides using *in silico* approach (**A**) Schematic diagram of human-derived penetrating peptide identification. (**B**) SVM score of the potential CPPs from SwissProt database. Black, red and yellow color bars represent hPP3, hPP10 and hPP33 respectively. (**C**) Cellular uptake of FITC-labeled peptides (hPP3, hPP10 and hPP33) as determined by fluorescence microscopy. RFI: Relative fluorescence Intensity.

Successful prediction of effectiveness of CPPs using computational approach can significantly accelerate the selection of peptides for chemical synthesis to experimentally verify their cell penetrating potential, although this prediction is not yet a routine task. It has previously been shown that most of CPPs consist of multiple Arg residues, therefore, we considered it's likely that many arginine-rich CPP of SwissProt proteins should have cell penetrating properties [5, 38, 39]. In order to identify new and efficient human-derived CPP candidates, we have screened all possible arginine-rich sequences in SwissProt proteins (Figure 1A). Peptides with RXXRXX, XRXXRX or XXRXXR (X: R/K) motif, non-redundant, without Asp or Glu residue, non-C-terminus, containing 10 or 11 Arg/Lys of window length 20 were searched in SwissProt proteins. As a result, 33 sequences (summarized in Supplementary Table S1) satisfying the above mentioned criteria were found (Figure 1A), and these peptides were submitted to CellPPD webserver to predict. CellPPD can be used to predict highly efficient CPPs and help us to find novel CPP more speedily and conveniently. Higher the SVM score indicated that a given sequence will be higher probability to be a CPP (Figure 1B). Based on SVM scores of these peptide, we selected hPP3 (low score), hPP10 (high score) and hPP33 (Asp residue) peptide for further analysis of penetration (Figure 1C), uptake of hPP10 was significantly higher than hPP3 and hPP33. Taken together, these results suggest that hPP10 may represent as a new and efficient penetrating peptide, and hPP10 were used for further analysis on its characteristic and cargo delivery potential *in vivo* and *in vitro*.

### Structure analysis of hPP10 peptide

**Figure 2:**
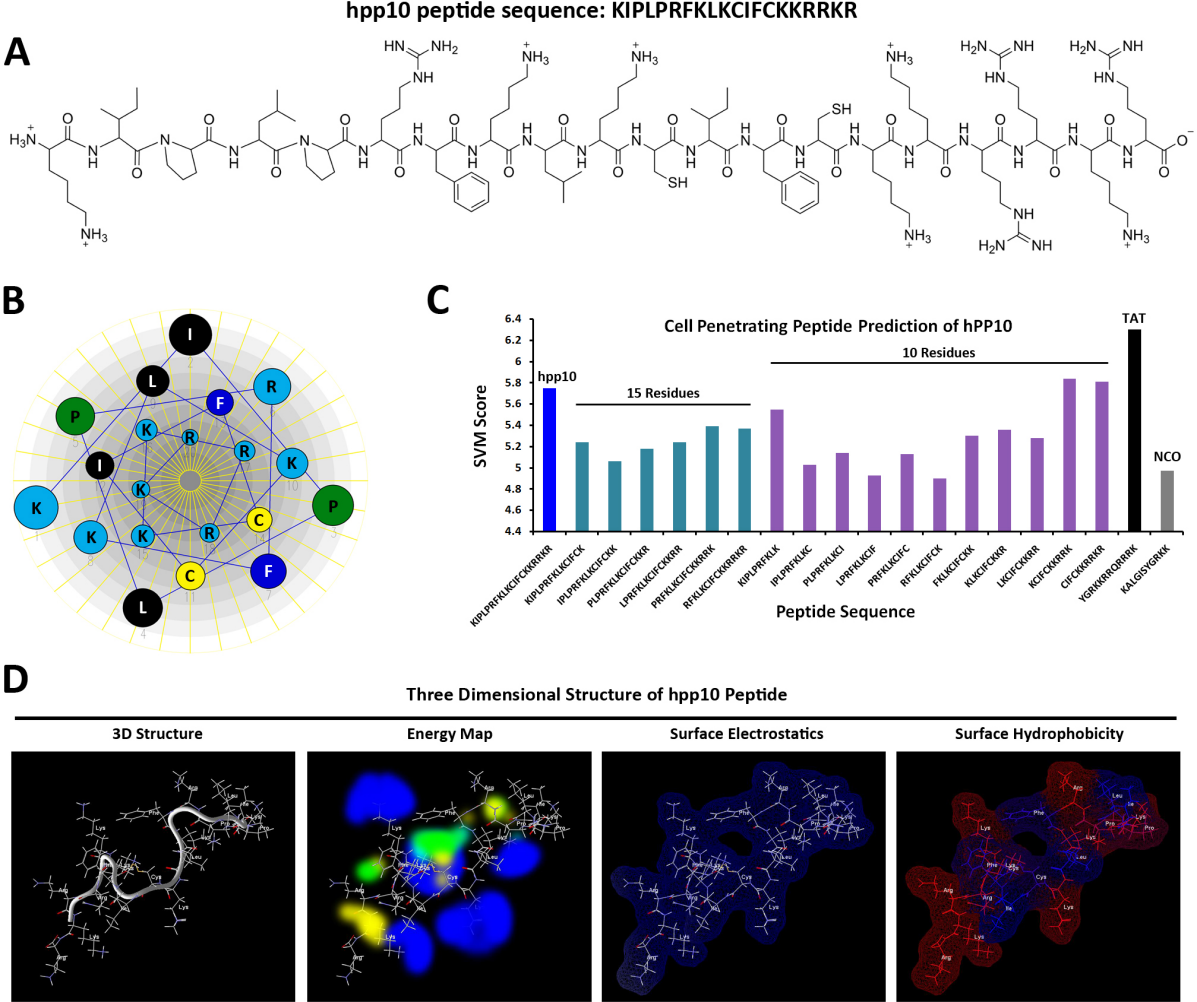
CPP prediction and three-dimensional (3-D) characterization of hPP10 (**A**) Amino acid sequence and primary structure of hPP10 peptide. (**B**) The Schiffer-Edmundson helical wheel representation of hPP10, helical wheel projects the arrangement of amino acids, and residue numbers are counted from the amino (N) terminus of hPP10. (**C**) SVM score of the CPP predicted motifs, different color bars represent full length of hPP10, 15 and 10 amino acid fragments, as well as TAT and NCO, respectively. (**D**) 3-D structure, energy map (blue color represents hydrogen acceptor favorable, green color represents steric favorable region, yellow color represents hydrogen donor favorable region associated with hPP10), surface electrostatics and surface hydrophobicity of hPP10.

KDM4A (also known as JMJD2A and JHDM3A) is the member of Jumonji domain 2 (JMJD2) histone demethylases family, which plays a critical role in genome stability and replication through the conversion histone H3 lysines 9 and 36 tri- and dimethylation (H3K9/36me3/2) to the demethylation status [34, 35]. Firstly, hPP10 (amino acids 820–839, located in the core region of the PHD domain, as is shown in Supplementary Figure S1A) from JMJD2A was identified and then characterized for its penetrating efficiency. Previous report have been shown that effective CPPs are known to possess a positive charge (Supplementary Figure S1B), and the arginine residues appear to contribute more to cellular internalization than lysine residues, amino acid composition and sequence arrangement of hPP10 was shown in Figure 2A. The physicochemical properties of hPP10, such as molecular weight, hydrophobicity, hydrophilicity, hydropathicity, amphipathicity, stearic hindrance, net hydrogen, side bulk, charge and pI were determined (Supplementary Table S2). The Schiffer-Edmundson helical wheel modeling with DNAstar program suggested the possible amphipathic α-helical conformation (Figure 2B). Furthermore, secondary conformations and solvent accessibility (NetSurfP web server), 3D predicted models (I-TASSER server) and Ramachandran plot (VADAR web server) analysis (Supplementary Figure S2) are developed. Prediction of hPP10 as a CPP along with all the possible 10 and 15 residue long motifs scanned within this peptide (Figure 2C). The three dimensional structure of hPP10 had an M-shape like structure, which displayed hydrogen exchange space, electrostatic surface distribution and hydrophobicity associated with the hPP10 (Figure 2D). Upon careful consideration of its properties *in silico* shown above, the hPP10 was functionalized onto different cargos for intracellular delivery.

### Cellular uptake of hPP10-FITC

**Figure 3:**
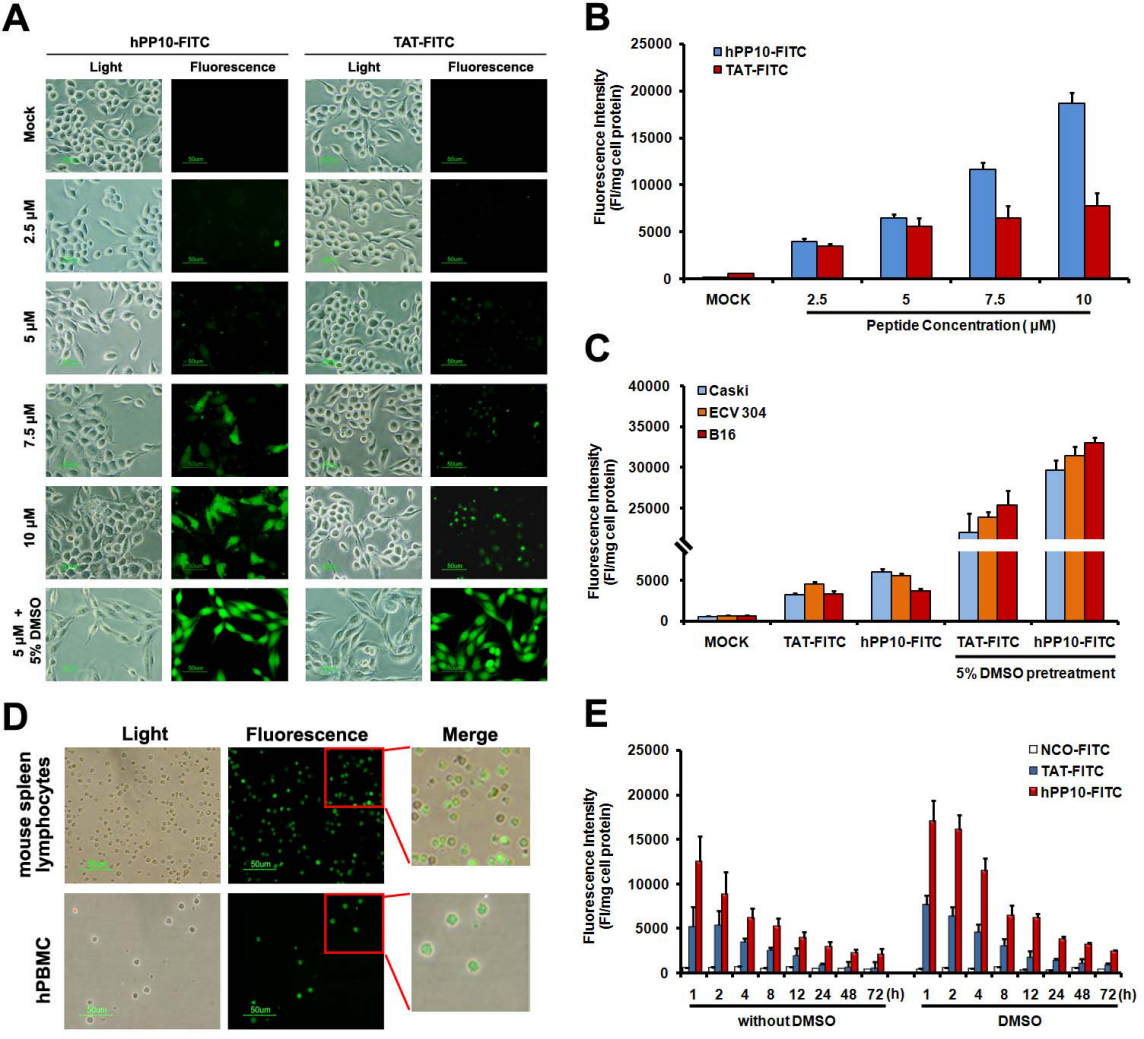
FITC-labeled peptides uptaken by Caski cell pretreated with different concentrations of DMSO (**A**) Cell-permeability of FITC-labeled hPP10 with its concentrations from 2.5 μM to 10 μM in Caski cells, hPP10-FITC was well-distributed in Caski cells observed under fluorescence microscopy, and TAT-FITC was used as the positive control, scale bars, 50 μm. (**B**) Fluorescence intensity quantitation of FITC-labeled hPP10 uptaken in Caski cell. (**C**) Fluorescence intensity of FITC-labeled hPP10 uptaken in different cells by incubation with 5 μM FITC-labeled peptides for 1 h. (**D**) Cell-permeability of FITC-labeled hPP10 in primary cultured mouse spleen lymphocyte and human peripheral blood lymphocytes (hPBMCs) with 5% DMSO treatment, scale bars, 50 μm. (**E**) The long-lasting (1 to 72 h) fluorescence of FITC-labeled hPP10 in Caski cells with or without DMSO treatment.

To experimentally validate the predicted hPP10 have cell-penetrating capability, the uptake of synthesized FITC-labeled hPP10 was investigated (FITC-labeled TAT used for positive control), as viewed by fluorescence microscope. As is shown in Figure 3A, both of hPP10-FITC and TAT-FITC were taken up by Caski cells with same condition, localizing to both the cytoplasm and nucleus, however, the fluorescence of cytosol in hPP10 group was higher than TAT-FITC group. Our previous study have shown that 10% DMSO enhanced the penetrating efficiency of TAT, whether the penetration of hPP10 could be enhanced is still unknown, thus, we observed the fluorescence distribution of hPP10 in the presence of DMSO treatment, based on the present data we have obtained, which means that the uptake of hPP10 also could be effectively promoted by DMSO treatment. Penetrating efficiency of truncated hPP10 was also evaluated (Supplementary Figure S3), however, there was no significant difference between truncated hPP10-1, hPP10-2 and hPP10-3 in different cell lines with or without DMSO. The intracellular fluorescence intensity result shown in Figure 3B was consistent with fluorescence observations. Subsequently, we found that hPP10 was capable of permeating a wide variety of cancerous or immortalized cell lines, including B16 and ECV304 (Figure 3C), HepG2, T24, L929, HSC-T6 and THP1 (Supplementary Figure S2), hPP10 was able to permeate into primary cultured cells, such as mouse spleen lymphocytes, human peripheral blood lymphocytes (Figure 3D) and mouse primary fibroblast cells (Supplementary Figure S4). Moreover, we also found that hPP10-FITC was long-lasting fluorescence than TAT-FITC with or without DMSO treatment in Caski cells (Figure 3E).

**Figure 4:**
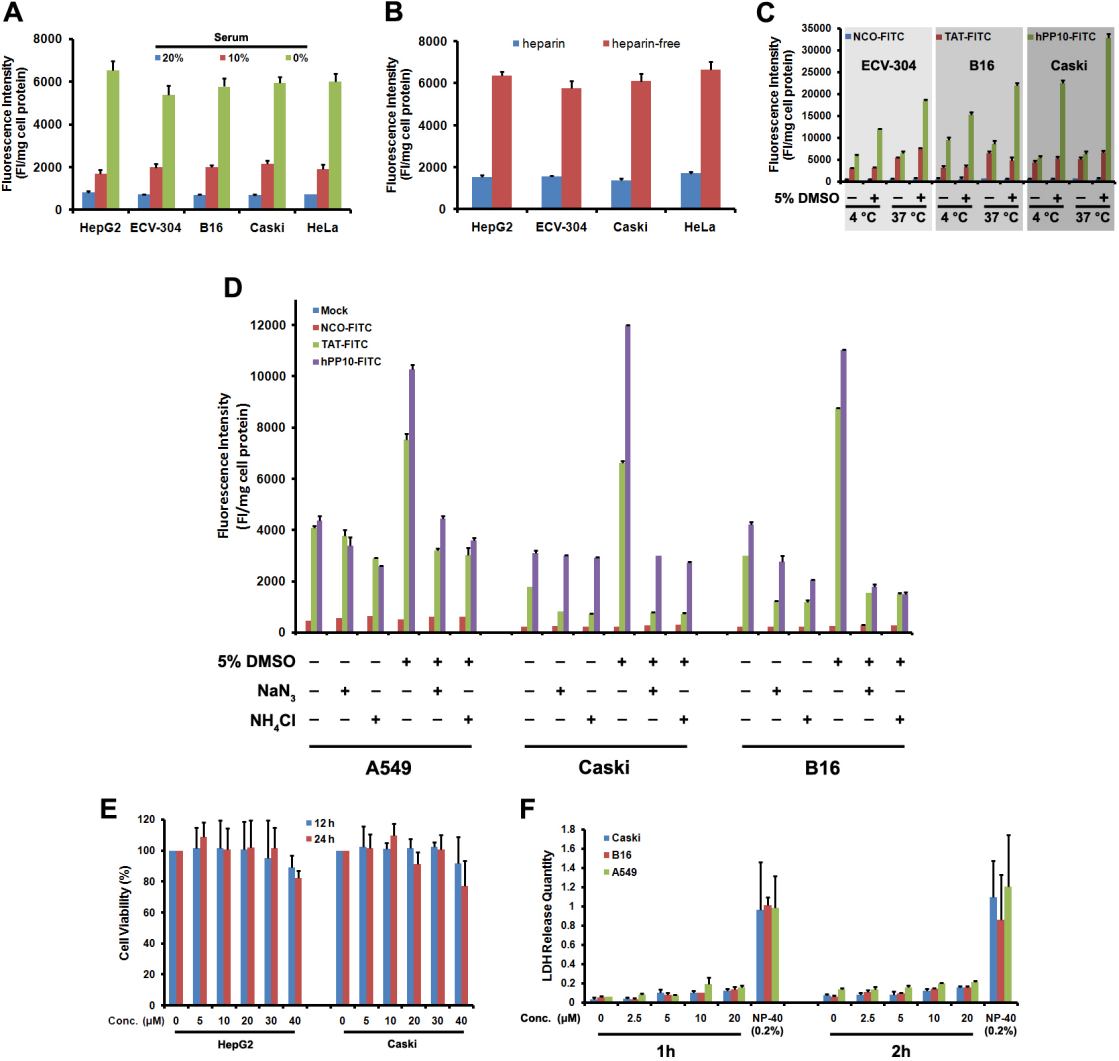
Effects of serum, heparin and temperature on uptake of hPP10 and its safety evaluation (**A**) Effects of serum on uptake of hPP10 in different cell lines; Error bars represent the mean ± SD for 3 samples. (**B**) Effects of heparin on cellular uptake of hPP10. (**C**) Effects of temperature on uptake of hPP10. (**D**) Suppressive effect of NaN3, NH4Cl exposure on hPP10-FITC uptake. (**E**) Different cells' viability evaluated by MTT assay. (**F**) LDH release quantity was determined after different concentrations of hPP10, positive control using 0.2% NP-40.

Previous results [36, 37] have shown that transduction efficiency of CPP is correlate with culture conditions, such as serum concentration, similarly, we also found that penetrating efficiency of hPP10-FITC decreased in the presence of serum (Figure 4A), this has been possible due to the CPP inactivation by serum proteases or special proteins. At present, the exact mechanism (endocytosis and direct translocation) used by CPPs to enter into the cellular is still debated [1, 25], ligands that interact with proteoglycans can trigger uptake through an endocytotic pathway [1]. To reveal the internalization process or mechanism, we analyzed the effects of heparin, temperature and endocytosis inhibitor on the cellular uptake of hPP10. When different cells were treated with hPP10-FITC co-incubated with 0.5 U/mL heparin, cellular uptake of hPP10-FITC was greatly decreased in the presence of heparin (Figure 4B). After incubation of the cells at 4°C or 37°C (Figure 4C), the efficiency of internalization were different between TAT-FITC and hPP10-FITC (cellular uptake of TAT-FITC was energy-dependent with or without DMSO treatment, while hPP10-FITC uptake was less energy-sensitive than that of TAT-FITC without DMSO treatment, but similar of energy-dependent pathway incubated with DMSO). Similar results observed for the effects of endocytosis inhibitors NaN_3_ (oxidative phosphorylation inhibitor as energy depleter [23]) and NH4Cl (anti-acidification agent as endocytic inhibitor [23]) on cellular uptake (Figure 4D). Subsequently, the safety or cytotoxicity of hPP10 was evaluated through MTT assay (Figure 4E) and LDH release assay (Figure 4F). There was minimal cytotoxicity (little repressive effect or membrane disturbance) to different cell lines with different concentration of hPP10. These results here suggested that uptake of hPP10 is associated with low membrane disturbance or detectable cytotoxicity through energy-dependent pathway.

### Cellular uptake of hPP10-GFP or apoptin fusion protein

**Figure 5:**
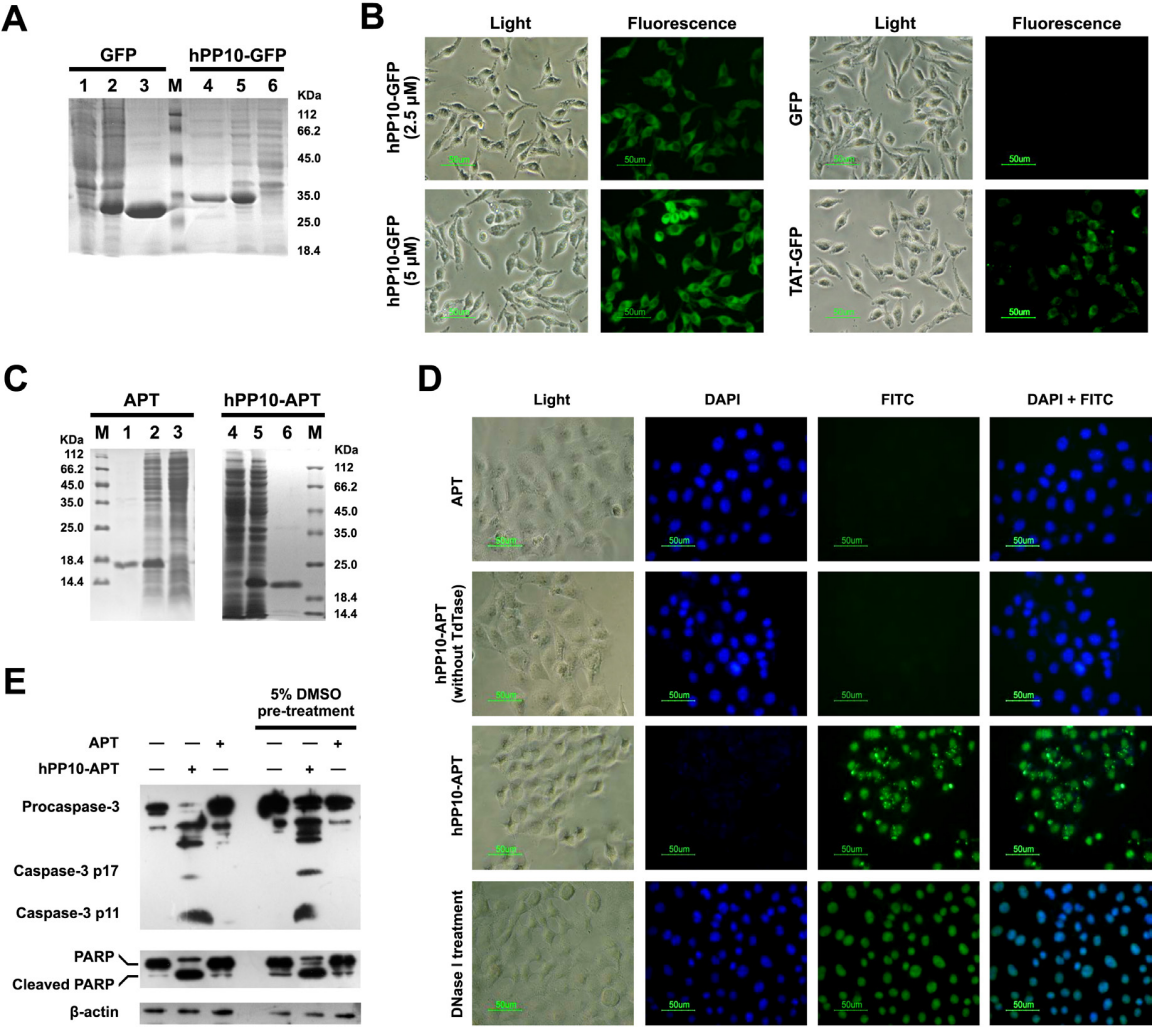
Fusion protein (hPP10-GFP and hPP10-Apoptin) penetration and functionalization *in vitro* (**A**) Recombinant GFP and hPP10-GFP protein expression detected by Coomassie Brilliant Blue staining. Lane 1 and 6: without IPTG induction, lane 2 and 5: IPTG induction, lane 3 and 4 purified fusion protein. (**B**) TAT-GFP AND hPP10-GFP fusion proteins' transduction ability detected by under fluorescence microscopy. (**C**) Recombinant Apoptin and hPP10-Apoptin protein expression and purification. Lane 1 and 6: purified fusion protein, lane 2 and 5: IPTG induction, lane 3 and 4 without IPTG induction. (**D**) TUNEL assay of B16 melanoma cells apoptosis after treating by fusion protein. Nuclei were counterstained with DAPI. (**E**) Western blotting assay on apoptotic relative protein, including Caspase-3 and PARP.

In order to assess the penetrating capability of hPP10 fused with functional macromolecules, recombinant hPP10-GFP protein (Figure 5A) was expressed and purified, and the subcellular distribution of hPP10-GFP in B16 cells was viewed by fluorescence microscope, as is shown in Figure 5B, GFP and TAT-GFP (5 μM) was used as control, fluorescent of hPP10-GFP was well-distributed in the cytosol of cells, hPP10-GFP fusion protein uptake efficiency was higher than TAT-GFP with DMSO treatment in ECV304 and Cos7 cells (Supplementary Figure S5), this result is consistent with FITC-labeled hPP10 peptide uptake. Recent studies have shown that Apoptin protein encoded by the Chicken Anemia virus (CAV), possess specifically ability to kill tumor cells [23, 38], while the family mitochondria disrupting peptides, KLA peptide have proapoptotic ability without cell type targeting [39]. Likewise, we fused Apoptin to the C-terminus of hPP10, and hPP10-Apoptin fusion protein was purified using the same procedure as for hPP10-GFP protein purification (Figure 5C). To prove that hPP10 can serve as a vehicle for functional cargo deliver *in vitro*, purified hPP10-Apoptin (5 μM) was used to treat cancer cell (B16 mouse melanoma cell line) or normal cell (L929 mouse fibroblastic cell line). From the fluorescence-based TUNEL assay, B16 (Figure 5D) cells apoptosis can be induced by hPP10-Apoptin and hPP10-KLA (Supplementary Figure S6A); however, no apoptosis was detected in L929 cells with hPP10-Apoptin treatment, while apoptotic L929 cell can be detected after hPP10-KLA incubation (Supplementary Figure S6B). Again, cleaved Caspase-3 and caspase-dependent Poly (ADP-Ribose) Polymerase (PARP) cleavage was detected by Western blotting analysis (Figure 5E).

**Figure 6:**
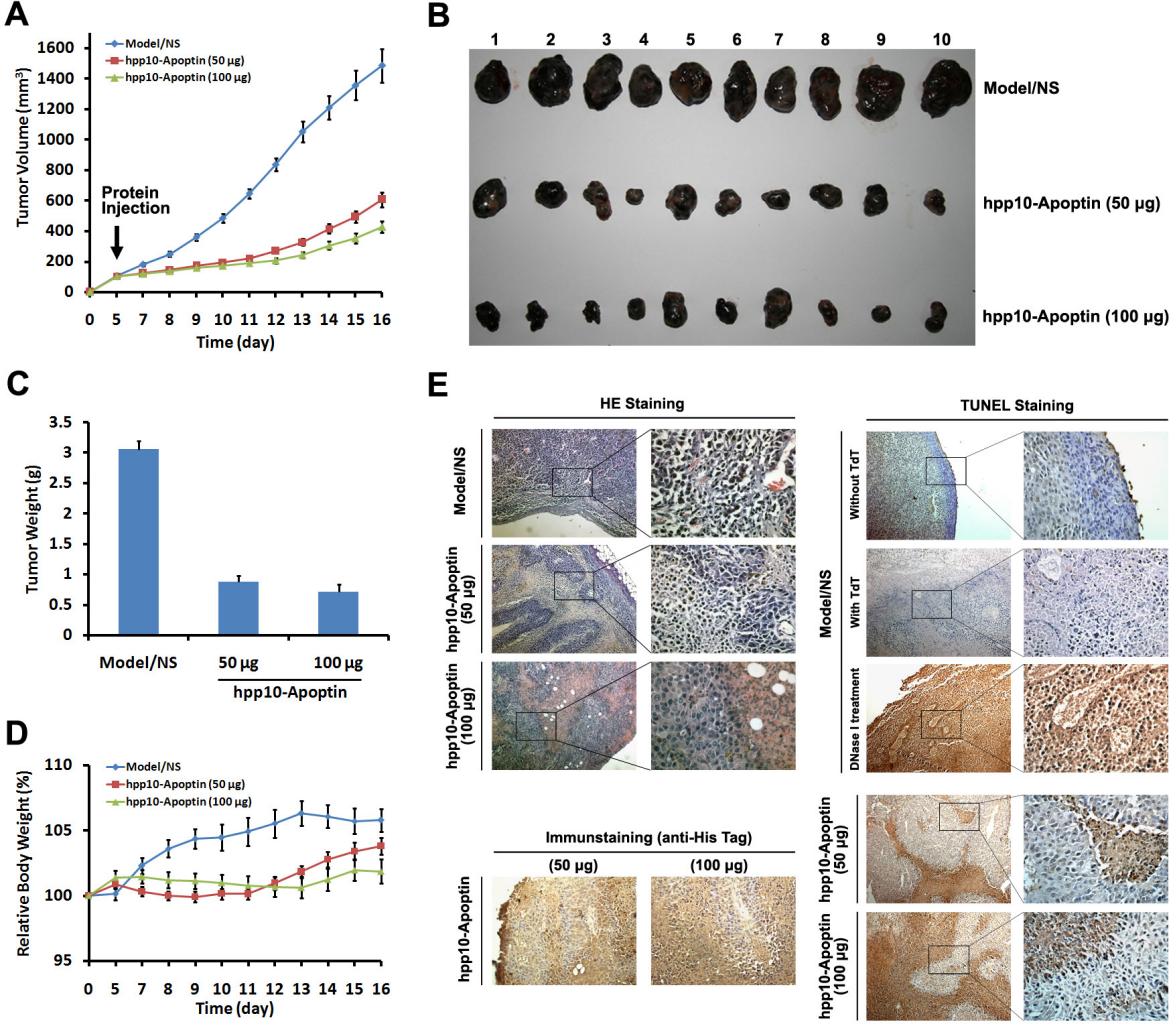
Tumor growth inhibition of hPP10-mediated Apoptin in B16 melanoma cell bearing mice *in vivo* (**A**) After the tumors had reached initial size ~ 100–150 mm^3^ (mean diameter of 8 to 10 mm), 50 or 100 μg of hPP10-Apoptin were administered, control animals (NS) received PBS injections only, xenografted tumor volume was determined *in vivo* by external caliper, data are presented as mean ± SE of 10 animals. (**B**) Photographs of isolated tumors (*n* = 10) after 16 days of treatment with hPP10-Apoptin. (**C**) Tumor weight of mice (*n* = 10) treated with hPP10-Apoptin fusion protein was significantly decreased. (**D**) Relative body weight of tumor-bearing mice (*n* = 6) treated with hPP10-Apoptin fusion protein. (**E**) H&E-staining images of representative specimens with different groups at ×100 magnification, hPP10-Apoptin distribution (anti-His staining) was detected by immunohistochemistry, and representative images show positive detection. TUNEL staining images of representative specimens at ×100 magnification.

Furthermore, anticancer effects of hPP10-Apoptin were examined under *in vivo* condition. Different amount of hPP10-Apoptin fusion protein was injected into C57BL/6 mice bearing subcutaneous melanoma tumors for 5 days (Figure 6A), the tumors volume based on caliper measurements were done every day and the excised tumors of each treated group are shown in Figure 6B. The tumor weights of hPP10-Apoptin (50 μg and 100 μg) group were significantly decreased compared with PBS control group (Figure 6C), while, there is not significance between 50 μg and 100 μg of hPP10-Apoptin (Figure 6D). To analyze the contribution of hPP10-Apoptin fusion protein induction of tumor growth inhibition, we determined the hPP10-Apoptin could penetrate into melanoma tumor tissue and hPP10-Apoptin can induce apoptosis using H&E staining, immunohistochemistry and TUNEL assay (Figure 6E). Thereby, the present results have shown that functional GFP and apoptotic induction protein-Apoptin can be delivered by hPP10.

### Anti-hepatic fibrosis effect of hPP10-GCLC

**Figure 7:**
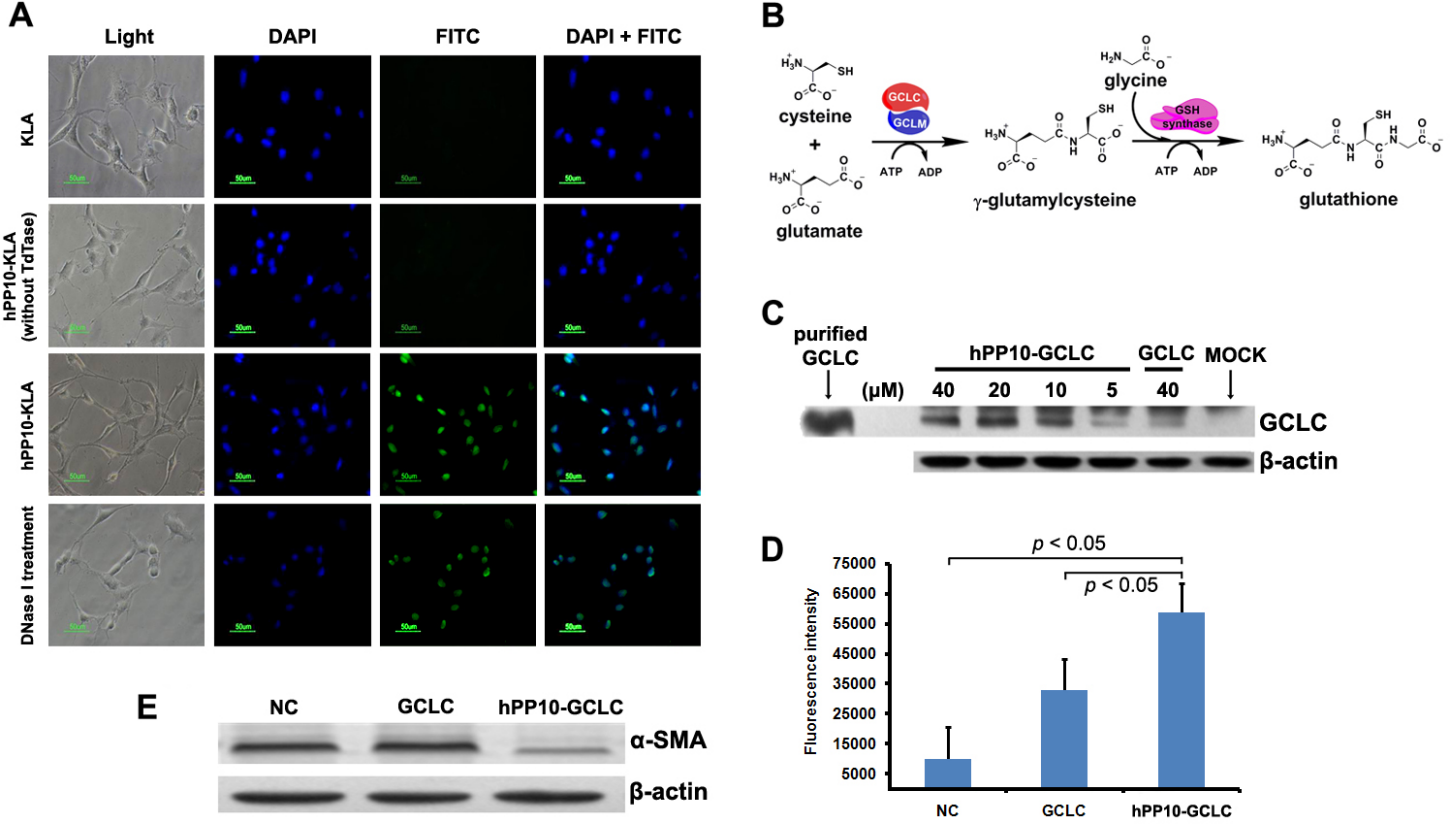
Efficient transduction of short peptide or protein in fibrosis related HSC-T6 cells *in vitro* (**A**)Apoposis induced by after hPP10-KLA incubation in HSC-T6 cells, TUNEL assay was performed for apoptosis detection. (**B**) Schematic diagram of the glutathione biosynthesis biological process. (**C**) After treating with different dosage of hPP10-GCLC fusion protein on HSC-T6 cells for 1 h, intracellular localization of hPP10-GCLC were detected by Western blotting, purified GCLC was used as postive control. (**D**) Intracellular glutathione (GSH) was detected using GSH-Glo^™^ Glutathione Assay. (**E**) After incubated with hPP10-GCLC on HSC-T6 cells, alpha-SMA expression was analyzed by Western blotting.

We studied the effect of hPP10-KLA on HSC-T6 cells apoptosis (Figure 7A), the above data demonstrate that hPP10 can be served as new tool for anti-fibrosis therapy. Furthermore, glutathione (GSH) is a major intracellular non-enzymatic antioxidant in mammals, and the liver is the major site of its synthesis and the major storage organ for GSH [40]. It is well known that hepatic oxidative stress is associated with hepatic fibrosis. Recent reports have shown that GSH levels are depleted in hepatic and lung fibrosis [41, 42], thus, we hypothesized that GSH supplement can revert activated hepatic stellate cell (HSC) back to quiescent state. The first rate-limiting enzyme of glutathione synthesis (Figure 7B), γ-glutamylcysteine synthetase (also known as Glutamate-cysteine ligase) is composed of catalytic subunit (GCLC) and regulatory subunit (GCLM). A key question was whether large therapeutic cargo such as enzyme could be delivered by hPP10. Recombinant rat GCLC expressing vectors were constructed and hPP10-GCLC fusion protein was purified (Supplementary Figure S7). From the Western blotting assay (Figure 7C), we found that hPP10-GCLC penetration into intracellular of cultured HSC-T6 cells was concentration-dependent, and further experiments using GSH-Glo^™^ Glutathione Assay to determine the intracellular GSH level, which can be used to evaluate the GCLC enzymatic activity, the present data showed that GCLC delivered into cells is biologically active (Figure 7D), thus suggesting that GCLC can be efficiently delivered into HSC-T6 cells by hPP10. Moreover, the anti-fibrosis potential of hPP10-GCLC was evaluated. In HSC-T6 cells, Western blotting assay revealed that significant suppression of α-SMA (Figure 7E) after hPP10-GCLC treatment.

**Figure 8:**
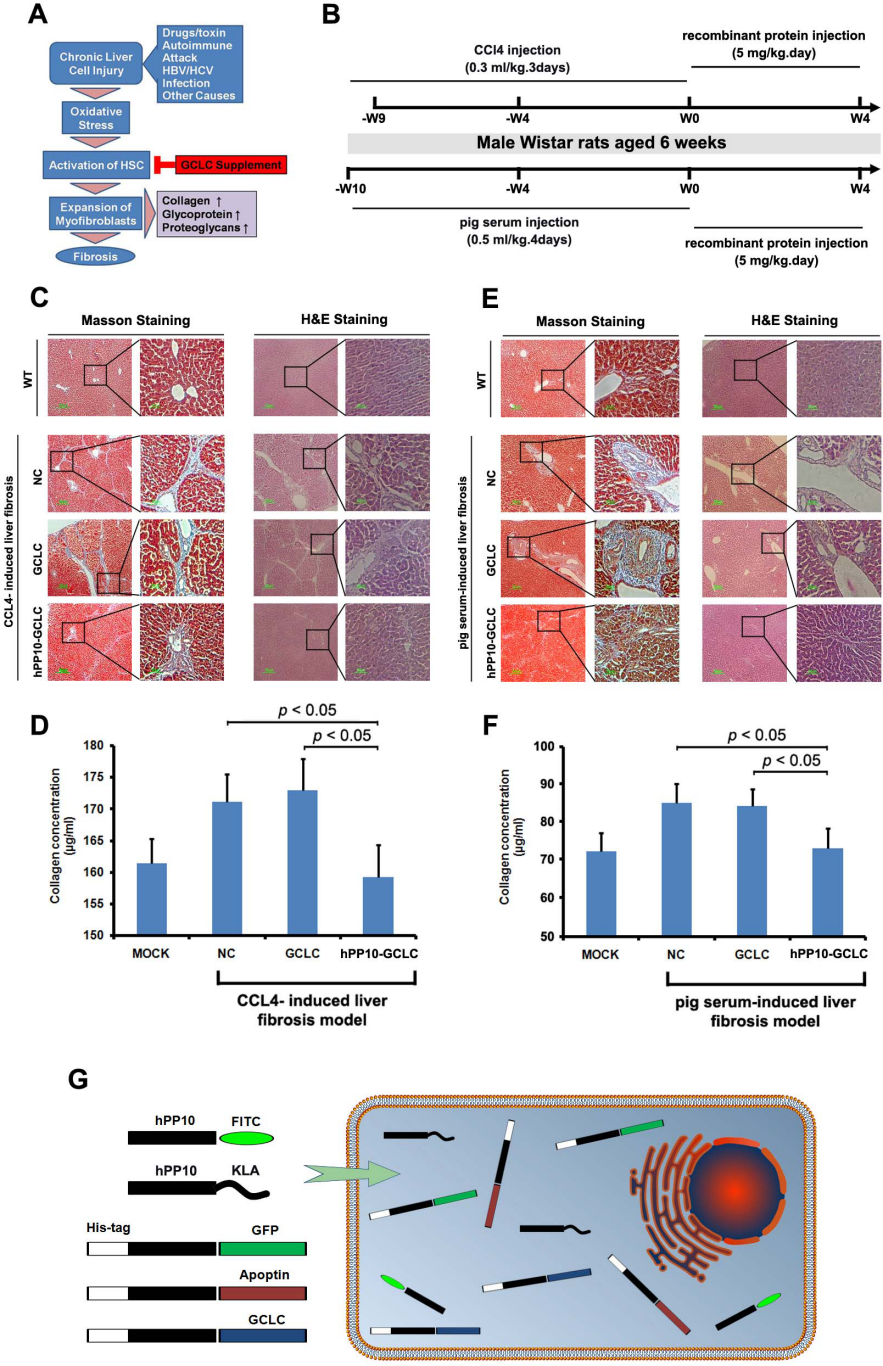
Anti-liver fibrosis effect of hPP10 conjugated with GCLC (**A**) Schematic diagram of the GCLC supplement to suppress the hepatic fibrosis. (**B**) Schematic timeline representation of rat model preparing and recombinant protein injection for anti-liver fibrosis therapy. (**C**) Masson staining was used to detect the collagen with or without hPP10-GCLC therapy on CCL4-induced liver fibrosis model, following routine histopathological examination with HE staining was performed. PBS-inoculated C57BL/6 rat liver fibrosis model was used as negative control. (**D**) The extent of total collagen in CCL4-induced liver fibrosis rat models was evaluated. (**E**) Masson staining was used to detect the collagen with or without hPP10-GCLC therapy on pig serum-induced liver fibrosis rat model, following routine histopathological examination with HE staining was performed. PBS-inoculated C57BL/6 rat liver fibrosis model was used as negative control. (**F**) The extent of total collagen in pig serum-induced liver fibrosis rat models was evaluated. (**G**) Human-derived penetrating peptide serves as an efficient tool for peptides or native functional proteins delivery into cultured cell lines or primary cells.

Expression of many antioxidant proteins such as glutathione S-transferases (GSTs), and glutamate-cysteine ligase catalytic subunit (GCLC), can lead to a rapid recovery of glutathione levels (Figure 8A), which may represent a useful strategy to prevent liver damage after liver fibrosis stimulators insults. To establish fibrosis of rat model, CCL4 or pig serum, as well as recombinant protein used later on was injected following the timeline shown in Figure 8B. *In vivo*, Masson staining (Figure 8C) and quantification of collagen (Figure 8D) evaluation showed hPP10-GCLC treatment significantly decreased collagens content in rat model, including CCL4-induced liver fibrosis (Figure 8C and 8D) and pig serum-induced liver fibrosis model (Figure 8E and 8F).

## DISCUSSION

Although a variety of tools have been developed as candidates for drug delivery [43–45], CPPs have great potential for therapeutic use, translocating attached cargos (e.g., proteins, genes, nanoparticles) [5, 10], as well as non-viral and/or integration-free method to generate induced pluripotent stem cells (iPSCs) through CPPs tagged reprogramming factors mediated somatic cells reprogramming [5, 17, 46–48], as well as CPP-based method that rapidly and reversibly endogenous gene knock down [49] or knock out [19].

Here, we investigated that hPP10 (represents a new class of CPP) add to a growing list of translocating sequences that enhance functionalized protein and enzyme for intracellular delivery into cultured cells, as well as mouse and rat model. The hPP10 described in present study were characterized according to cell-penetrating properties which have positively charged arginine and lysine residue, it bears resemblance to previous described CPPs [50–55]. *In vitro* assay, hPP10 peptide can be taken up not only by various cultured cell lines associated with low membrane disturbance or detectable cytotoxicity through energy dependent pathway, but also primary cell cultures *in vitro*, localizing to both the nucleus and cytoplasm as shown by fluorescence microscopy. In our previous study, we identified several small molecule [23, 24] can enhance the penetrating efficiency of CPP, our results in present is consistent with our previous studies in that hPP10 can enter cells in a highly efficient manner after DMSO treatment. To further evaluating the effectiveness of a potential delivery tool hPP10 for other macromolecules, GFP and Apoptin were conjugated with the C-terminal fragment of hPP10 separately, and their fusion protein produced using prokaryotic expression systems, then we directly visualized the well-distributed in cellular of these conjugated protein. Furthermore, we evaluated the function of these cargo, including the apoptosis induction by Apoptin, although the exact mechanism of apoptosis induced by Apoptin remains obscure [56]. Apoptin fused with hPP10 penetrate into cytoplasm and induce the cancer cell apoptosis. Thus, in summary, these functional protein cargos' uptake could be helped by hPP10 (Figure 8G).

Several reports implicated knockdown of GCLC exacerbated bile duct ligation-induced liver injury and fibrosis [41], which suggested that cellular GSH supplement provide a novel means to suppress hepatic fibrosis. Although previous study has shown that wild-type or dominant-inhibitory mutants of the GCL subunits by TAT transduction is able to manipulate cellular GCL activity [57], whether anti-fibrosis effect in HSCs can be generated through suppression of oxidative stress mediated by GCLC supplement remains unknown. From our present study, GCLC conjugated with hPP10 markedly decrease the processes that lead to liver fibrosis through the suppression of liver-related genes, especially collagen and α-smooth muscle actin (α-SMA). In this regard, hPP10-GCLC could be employed to rescue the hepatic fibrosis.

The immunogenicity of nonhuman originated CPPs carry the risk of possible cytotoxicity and immunogenicity, and human-derived CPPs enable decrease the risk of immunogenicity when they used *in vivo* applications further. Although the mechanism of hPP10 penetration is still obscure, our results shown that hPP10 may penetrate into cultured cells through a partial endocytic pathway. More importantly, we can use intermolecular crosslinking between the two cysteine amino acid residues to make the hPP10 more stable. Taken as a whole, we have developed a novel; human-derived safe and less antigenicity of cell-permeable peptides that allow exogenous bioactive molecules intracellular delivery efficiently, therefore, the unique characteristics of hPP10 make it a promising approach to deliver drugs or reprogrammed cell-based therapy.

## MATERIALS AND METHODS

### hPP10 characterization

Non-cell-penetrating or cell-penetrating peptide analysis with bioinformatic approach would accelerate its identification, hPP10 derived from KDM4A protein was first predicted, and then evaluated by prediction tools for cell penetrating efficiency. The support vector machine (SVM)-based CellPPD web server was used for rapid screening of CPP and its efficient evaluation [58]. The Motif-based method and SVM-based method were used to predict (SVM threshold of – 0.1 and a motif e-value of 10). The 3D (three dimensional) structure of hPP10 was analyzed using peptide tertiary structure prediction server (Pepstr), which allows modeling of small peptides with sequence length varying between 7 to 25 residues [59]. Helical properties of hPP10 were delineated with Schiffer Edmundson wheel modelling using DNASTAR Lasergene 7 programme. We also predicted the 3D structure of hPP10 peptide using I-TASSER program (http://zhanglab.ccmb.med.umich.edu/I-TASSER). The modeled structure is refined with molecular dynamic simulations and energy minimization using Pepstr server, VADAR (Volume Area Dihedral Angle Reporter) web server was used to validate the structure [60], the surface electrostatics and energy map of the peptide were analyzed with Molegro molecular viewer.

### Cells, peptides and proteins

The human A549 (lung cancer), HepG2 (liver cancer), Caski and HeLa (cervical cancer), ECV-304 (endothelial cells), mouse B16 (melanoma) and rat HSC-T6 (hepatic stellate cell) cell lines were maintained in our laboratory. These cell lines were cultured with RPMI 1640 medium (Invitrogen, USA) supplemented with 10% FBS (Invitrogen, USA) at 5% CO_2_ and 37°C. Primary cells were isolated from CD1 strain mouse.

All soluble FITC-labeled peptides (hPP10: KIPLPRFKLKCIFCKKRRKR, TAT: YGRKKRRQ RRRK; NCO, a nonsense peptide: KALGISYGRKK; KLA: KLAKLAKKLAKLAK, and NBD: GGTA LDWSWLQTE) were obtained from SBS Genetech (Beijing, China). Peptides were synthesized using the standard Fmoc-chemistry-based strategy, purified and analyzed by reversed-phase high performance liquid chromatography (RP-HPLC) to more than 99% purity, diluted to 500 mM in PBS, and then stored at −20°C for further use.

TAT-GFP and GFP protein were prepared following the protocol described [23, 24], nucleotide sequence of the hPP10 was available in GenBank (NM_014663.2), hPP10-GFP, hPP10-Apoptin, hPP10-GCLC DNAs were constructed into pET15b plasmid and recombinant fusion proteins were purified in the BL21 (DE3) strain of *E. coli*. Briefly, after the constructed plasmids were cloned into pET15b vector, *E.coli* BL21 (DE3) star pLysS cells were transformed with these plasmids. Colonies were inoculated into 50 ml of Luria-Bertani (LB) media containing ampicillin (50 μg/ml) and grown for 12 h at 37°C, 50 ml cultures were transferred into 500 ml of fresh LB medium and grown at 37°Cfor 1 to 2 h. Protein expression was induced by 1 mM isopropyl-β-D-thiogalactopyranoside (IPTG) at 37°C for 4 hs. Bacterial cells were harvested by centrifugation, and 6-His-tagged target proteins were purified through Ni-NTA affinity chromatography (Qiagen) and desalted using a column (Amersham).

### Cellular uptake studies

The internalization of FITC conjugated hPP10 into cultured cells was observed using fluorescence microscopy. All types of cells were plated in 24-well-plates (Greiner, Germany) and cultivated to semi-confluence in RPMI-1640 medium in a CO_2_ incubator for 24 h at 37°C. Cells were washed with PBS, followed by 5.0 μM FITC-labeled peptide or fusion protein incubation with serum-free medium for 1 h at 37°C, the medium was removed, the cells were further washed with PBS 3 times, and then imaged using fluorescence microscope (Nikon, Japan) with a band-pass filter.

Intracellular fluorescence intensity quantification was measured using Multimode Microplate Reader (Tecan 2000; Tecan, Mannedorf, Switzerland). The intracellular uptake of FITC-labeled peptide was performed as described previously [23]. Briefly, after the incubation, cells were washed and then lysed by adding 300 μl lysing buffer (0.1 M NaOH) for 10 min, the cell lysates were harvested and centrifuged (14000 g for 5 min), the supernatant was used to measure the fluorescence intensity. The fluorescence of cellular uptake is expressed as fluorescence intensity per mg of total cellular protein. All experiments were repeated at least three times and always performed in triplicate.

### *In vitro* cytotoxicity assays

### MTT assay

MTT assay was used to evaluate the cytotoxicity of hPP10 against different cell lines after short term of incubation. Cells were seeded in 96-well plates at 24 h before treatment. After being washed 3 times with PBS, cells were exposed to serum-free medium with different concentrations of hPP10, incubated for 12 h and 24 h and washed with PBS. MTT solution (5 mg/mL) was added into fresh serum medium for further 4 h incubation. DMSO was used to solubilize formazan, and the absorbance was measured using a Multiskan Spectrum plate reader (Thermo, USA).

### LDH (lactate dehydrogenase) leakage assays

Membrane integrity was assessed by lactate dehydrogenase (LDH) leakage into the culture medium. After cells seeded in 96-well plate for 24 h before treatment, various concentrations of hPP10 were added into culture medium for different time. Cell-free supernatant was obtained and transferred to another 96-well-plate; substrate mixture was added to each well for another 10 min enzymatic reaction. Absorbance was measured at 560/590 nm using a Multiskan Spectrum plate-reader (Thermo, USA).

### Functional assay of hPP10-linked cargos

### Western blotting

Western blot analysis was performed following the protocol described previously [24]. Briefly, after peptide or fusion protein treatment, cells were collected, washed and then pelleted by centrifugation. Cell pellets were lysed with RIPA buffer (150 mM sodium chloride, 1% Triton X-100, 1% sodium deoxycholate, 0.1% SDS, 50 mM Tris–HCl, pH 7.5, and 2 mM EDTA) with protease inhibitors. Total protein extracts were separated by sodium dodecyl sulfate polyacrylamide gel electrophoresis (SDS-PAGE) using a 10% SDS-PAGE gel, and then transferred onto a polyvinylidene difluoride (PVDF) membrane. PVDF membrane was blocked with blocking buffer (5% non-fat dry milk in 1× TBST buffer (0.1% Tween-20 in TBS)) 1 h at room temperature. Primary antibodies used for immunoreactions: anti-α-SMA (Rabbit polyclonal, Santa Cruz Biotechnology; 1:1000), anti-PARP (Mouse monoclonal, Santa Cruz Biotechnology; 1:1000), caspase-3 antibody (Rabbit, Cell signaling 1:1000), anti-His antibody (Rabbit, Cell signaling 1:1000). After rinsing the PVDF membrane, followed by incubation with horseradish peroxidase conjugated secondary antibody (Santa Cruz Biotech 1:5000) for 1 h at room temperature. Immunoreactions were also performed using β-actin antibody as loading controls. Signals were detected by chemiluminescence using an enhanced chemiluminescence (ECL)-detecting reagent.

### Animal model preparation and histological analysis

All experiments in mice were performed following the relevant institutional guidelines and regulations, and were subject to a protocol approved by the China Three Gorges University Animal Care Committee.

For melanoma-bearing mouse model generation, the B16 mouse melanoma cell line was subcultured twice and injected interdermally into the dorsal skin of each C57BL/6 mouse (5.0 × 10^4^ cells per injection). After 5 days growing, a palpable tumor had formed, following daily subcutaneous injection of hPP10-Apoptin (50 μg and 100 μg), and 16 days after injection, mouse was sacrificed and tumors were isolated, fixed in 10% neutral-buffered formalin, paraffin-embedded, and processed for H&E and immunohistochemical staining.

For CC_l4_-induced liver fibrosis model preparation, CC_l4_ was intraperitoneally administrated to rat at 0.3 ml/kg body weight for 9 weeks to induce rat (*n* = 24) live fibrosis [61]. For pig serum-induced rat liver fibrosis, rat (*n* = 24) were given intraperitoneal injections of 0.5 ml of normal pig serum twice a week for 10 weeks [62]. In rat model of liver fibrosis, hPP10-GCLC fusion protein (5 mg/kg) was administered intravenously via the tail vein and the animals were monitored for 3 weeks. Rat (*n* = 8) were injected with PBS to serve as controls. Rats were sacrificed after termination of the fusion protein treatment, liver tissue were removed and postfixed in 4% paraformaldehyde, and then performed following The H&E staining and the immunohistochemistry procedures.

### TUNEL analysis

Terminal deoxynucleotidyl transferase-mediated deoxyuridine triphosphate nick end labeling (TUNEL) staining was performed using fluorescein and/or horseradish peroxidase labeling *In Situ* Cell Death Detection Kit (Roche Applied Science, Germany) according to the manufacturer's protocol. The cell was incubated for 24 h in a fresh serum medium containing hPP10-Apoptin (5 μM), and then fixed with 1% paraformaldehyde in PBS, subjected to TUNEL staining. Cells were also stained with DAPI for 10 min and then rinsed with distilled water. A DAPI filter was used to detect DAPI staining (blue color), and an FITC filter was used was to detect TUNEL staining (green color). TUNEL-positive (green) and DAPI-positive (blue) staining patterns were acquired with fluorescence microscope (Nikon, Japan). TUNEL-positive cells in the different regions of each slide were counted by an observer who was blinded to the treatment conditions. While, sections were deparaffinized in xylene, rehydrated in decreasing concentrations of ethanol, digested in 0.5% pepsin for 60 min at 37°C, and endogenous peroxidase was blocked in 3% hydrogen peroxide. Terminal deoxynucleotidyl transferase (TdT) in reaction buffer was applied to serial sections for 1 h at 37°C. Following washes, a prediluted anti-digoxigenin peroxidase-conjugated antibody was applied for 30 min. After incubation in the 3,3′-diaminobenzidine (DAB) chromogen for approximately 6 min, apoptotic cells were visualized under microscopy with diaminobenzidine.

### Masson's trichrome staining and immunohistochemistry stains

Tissue sections were stained with Masson's trichrome for assessment of collagen fibers fibrosis and Hematoxylin-eosin (H&E) for morphological assessment. Masson's Trichrome staining was performed to detect collagen deposition as previously described [63]. For Masson's trichrome staining, the sections were deparaffinized and rehydrated through gradient alcohol, then sequentially treated with solution I (5% potassium dichromate and 5% TCA), Weigert's iron hematoxylin, solution II (1.25% phosphotungstic acid and 1.25% phosophomolybdic acid), 0.75% Orange G solution, solution III (0.12% xylidine Ponceau, 0.04% acid fuchsin, and 0.02% azophloxin), 2.5% phosphotungstic acid, and finally an aniline blue solution.

For H&E staining, tissue samples were fixed in 10% neutral buffered formalin and embedded in paraffin wax for H&E staining. Sections (4 μm thickness) were cut, dewaxed with xylene, hydrated through a series of ethanol washes and then stained. Alternatively, the de-paraffined sections were permeabilized by 0.2% Triton X-100 in PBS. And then they were incubated with anti-His antibody at 37°C for 1 h and subsequently with secondary antibodies for 1 h at 37°C. A streptavidin-enzyme conjugate was sequentially added for 20 min, and samples were incubated with substrate DAB, and then they underwent haematoxylin counterstaining. Negative control had no primary antibody.

### Total collagens analysis

Total collagens in the liver were examined by colorimetric determination of hydroxyproline residues by acid hydrolysis of collagens (Quickzyme, Leiden, Netherlands) according to the manufacturer's protocols. In brief, livers (5 mg) were acid hydrolyzed with 12 M HCl and incubated overnight at 95°C. The samples were then centrifuged; the total protein was quantified after supernatant collection. Samples were added to assigned wells in a 96-well plate, and the hydroxyproline content resulted in a chromogen with an absorbance maximum at 570 nm using a Multiskan Spectrum plate reader (Thermo, USA). The absolute collagen content was examined by comparison to the collagen standard provided by the kit.

### GSH assay

Intracellular GSH level in HSC-T6 cells was determined using GSH-Glo^™^ Glutathione Assay Kit (Promega, Madison, USA) according to the instructions. Briefly, HSC-T6 cells were cultured at 37°C in the presence or absence of hPP10-GCLC, washed with PBS. Subsequently, the GSH-Glo^™^ reagent was prepared according to the manufacturer's instructions, added to the wells and then incubated for 30 min at room temperature. Luciferin detection reagent was added to the well and then incubated for 15 min at room temperature. The levels of intracellular GSH were quantified using the GSH standards.

### Statistical analysis

All experiments were performed at least three times and all results are expressed as means ± standard deviation (SD). Statistical significance between groups was calculated using SPSS software. A student's *t*-test was used for data analysis and *p* value < 0.05 was taken as the level of statistically significant.

## SUPPLEMENTARY MATERIALS FIGURES AND TABLES





## References

[1] Copolovici DM, Langel K, Eriste E, Langel U (2014). Cell-penetrating peptides: design, synthesis, and applications. ACS Nano.

[2] Orange JS, May MJ (2008). Cell penetrating peptide inhibitors of nuclear factor-kappa B. Cell Mol Life Sci.

[3] Pae J, Pooga M (2014). Peptide-mediated delivery: an overview of pathways for efficient internalization. Ther Deliv.

[4] Koren E, Torchilin VP (2012). Cell-penetrating peptides: breaking through to the other side. Trends Mol Med.

[5] Liu H, Zeng F, Zhang M, Huang F, Wang J, Guo J, Liu C, Wang H (2016). Emerging landscape of cell penetrating peptide in reprogramming and gene editing. J Control Release.

[6] Milletti F (2012). Cell-penetrating peptides: classes, origin, and current landscape. Drug Discov Today.

[7] Walrant A, Bechara C, Alves ID, Sagan S (2012). Molecular partners for interaction and cell internalization of cell-penetrating peptides: how identical are they?. Nanomedicine (Lond).

[8] Yandek LE, Pokorny A, Floren A, Knoelke K, Langel U, Almeida PF (2007). Mechanism of the cell-penetrating peptide transportan 10 permeation of lipid bilayers. Biophys J.

[9] Morris MC, Deshayes S, Heitz F, Divita G (2008). Cell-penetrating peptides: from molecular mechanisms to therapeutics. Biol Cell.

[10] Wang F, Wang Y, Zhang X, Zhang W, Guo S, Jin F (2014). Recent progress of cell-penetrating peptides as new carriers for intracellular cargo delivery. J Control Release.

[11] Jo J, Hong S, Choi WY, Lee DR (2014). Cell-penetrating peptide (CPP)-conjugated proteins is an efficient tool for manipulation of human mesenchymal stromal cells. Sci Rep.

[12] Tiwari PM, Eroglu E, Bawage SS, Vig K, Miller ME, Pillai S, Dennis VA, Singh SR (2014). Enhanced intracellular translocation and biodistribution of gold nanoparticles functionalized with a cell-penetrating peptide (VG-21) from vesicular stomatitis virus. Biomaterials.

[13] Ma GS, Aznar N, Kalogriopoulos N, Midde KK, Lopez-Sanchez I, Sato E, Dunkel Y, Gallo RL, Ghosh P (2015). Therapeutic effects of cell-permeant peptides that activate G proteins downstream of growth factors. Proc Natl Acad Sci U S A.

[14] Erazo-Oliveras A, Najjar K, Dayani L, Wang TY, Johnson GA, Pellois JP (2014). Protein delivery into live cells by incubation with an endosomolytic agent. Nat Methods.

[15] Kim D, Kim CH, Moon JI, Chung YG, Chang MY, Han BS, Ko S, Yang E, Cha KY, Lanza R, Kim KS (2009). Generation of human induced pluripotent stem cells by direct delivery of reprogramming proteins. Cell Stem Cell.

[16] Zhou H, Wu S, Joo JY, Zhu S, Han DW, Lin T, Trauger S, Bien G, Yao S, Zhu Y, Siuzdak G, Scholer HR, Duan L (2009). Generation of induced pluripotent stem cells using recombinant proteins. Cell Stem Cell.

[17] Deng XY, Wang HU, Wang T, Fang XT, Zou LL, Li ZY, Liu CB (2014). Non-Viral Methods For Generating Integration-Free, Induced Pluripotent Stem Cells. Curr Stem Cell Res Ther.

[18] Zeng F, Huang F, Guo J, Hu X, Liu C, Wang H (2015). Emerging methods to generate artificial germ cells from stem cells. Biol Reprod.

[19] Ramakrishna S, Kwaku Dad AB, Beloor J, Gopalappa R, Lee SK, Kim H (2014). Gene disruption by cell-penetrating peptide-mediated delivery of Cas9 protein and guide RNA. Genome Res.

[20] Liu J, Gaj T, Patterson JT, Sirk SJ, Barbas CF (2014). Cell-penetrating peptide-mediated delivery of TALEN proteins via bioconjugation for genome engineering. PLoS One.

[21] D'Astolfo DS, Pagliero RJ, Pras A, Karthaus WR, Clevers H, Prasad V, Lebbink RJ, Rehmann H, Geijsen N (2015). Efficient intracellular delivery of native proteins. Cell.

[22] El-Andaloussi S, Jarver P, Johansson HJ, Langel U (2007). Cargo-dependent cytotoxicity and delivery efficacy of cell-penetrating peptides: a comparative study. Biochem J.

[23] Wang H, Zhong CY, Wu JF, Huang YB, Liu CB (2010). Enhancement of TAT cell membrane penetration efficiency by dimethyl sulphoxide. J Control Release.

[24] Ma JL, Wang H, Wang YL, Luo YH, Liu CB (2015). Enhanced Peptide Delivery into Cells by Using the Synergistic Effects of a Cell-Penetrating Peptide and a Chemical Drug to Alter Cell Permeability. Mol Pharm.

[25] Reissmann S (2014). Cell penetration: scope and limitations by the application of cell-penetrating peptides. J Pept Sci.

[26] Holton TA, Pollastri G, Shields DC, Mooney C (2013). CPPpred: prediction of cell penetrating peptides. Bioinformatics.

[27] Sanders WS, Johnston CI, Bridges SM, Burgess SC, Willeford KO (2011). Prediction of cell penetrating peptides by support vector machines. PLoS Comput Biol.

[28] Lindgren M, Langel U (2011). Classes and prediction of cell-penetrating peptides. Methods Mol Biol.

[29] Brock R (2014). The uptake of arginine-rich cell-penetrating peptides: putting the puzzle together. Bioconjug Chem.

[30] Bechara C, Sagan S (2013). Cell-penetrating peptides: 20 years later, where do we stand?. FEBS Lett.

[31] Rao Y, Kwok SJ, Lombardi J, Turro NJ, Eisenthal KB (2014). Label-free probe of HIV-1 TAT peptide binding to mimetic membranes. Proc Natl Acad Sci U S A.

[32] Tunnemann G, Ter-Avetisyan G, Martin RM, Stockl M, Herrmann A, Cardoso MC (2008). Live-cell analysis of cell penetration ability and toxicity of oligo-arginines. J Pept Sci.

[33] Gautam A, Sharma M, Vir P, Chaudhary K, Kapoor P, Kumar R, Nath SK, Raghava GP (2015). Identification and characterization of novel protein-derived arginine-rich cell-penetrating peptides. Eur J Pharm Biopharm.

[34] Berry WL, Janknecht R (2013). KDM4/JMJD2 histone demethylases: epigenetic regulators in cancer cells. Cancer Res.

[35] Marmorstein R, Trievel RC (2009). Histone modifying enzymes: structures, mechanisms, and specificities. Biochim Biophys Acta.

[36] Simon MJ, Gao S, Kang WH, Banta S, Morrison B (2009). TAT-mediated intracellular protein delivery to primary brain cells is dependent on glycosaminoglycan expression. Biotechnol Bioeng.

[37] Youngblood DS, Hatlevig SA, Hassinger JN, Iversen PL, Moulton HM (2007). Stability of cell-penetrating peptide-morpholino oligomer conjugates in human serum and in cells. Bioconjug Chem.

[38] Kim HY, Kim S, Youn H, Chung JK, Shin DH, Lee K (2011). The cell penetrating ability of the proapoptotic peptide, KLAKLAKKLAKLAK fused to the N-terminal protein transduction domain of translationally controlled tumor protein, MIIYRDLISH. Biomaterials.

[39] Ellerby HM, Arap W, Ellerby LM, Kain R, Andrusiak R, Rio GD, Krajewski S, Lombardo CR, Rao R, Ruoslahti E, Bredesen DE, Pasqualini R (1999). Anti-cancer activity of targeted pro-apoptotic peptides. Nat Med.

[40] Aquilano K, Baldelli S, Ciriolo MR (2014). Glutathione: new roles in redox signaling for an old antioxidant. Front Pharmacol.

[41] Ramani K, Tomasi ML, Yang H, Ko K, Lu SC (2012). Mechanism and significance of changes in glutamate-cysteine ligase expression during hepatic fibrogenesis. J Biol Chem.

[42] Liu RM, Vayalil PK, Ballinger C, Dickinson DA, Huang WT, Wang S, Kavanagh TJ, Matthews QL, Postlethwait EM (2012). Transforming growth factor beta suppresses glutamate-cysteine ligase gene expression and induces oxidative stress in a lung fibrosis model. Free Radic Biol Med.

[43] Choi JM, Ahn MH, Chae WJ, Jung YG, Park JC, Song HM, Kim YE, Shin JA, Park CS, Park JW, Park TK, Lee JH, Seo BF (2006). Intranasal delivery of the cytoplasmic domain of CTLA-4 using a novel protein transduction domain prevents allergic inflammation. Nat Med.

[44] Choi JM, Kim SH, Shin JH, Gibson T, Yoon BS, Lee DH, Lee SK, Bothwell AL, Lim JS, Lee SK (2008). Transduction of the cytoplasmic domain of CTLA-4 inhibits TcR-specific activation signals and prevents collagen-induced arthritis. Proc Natl Acad Sci U S A.

[45] Choi JM, Shin JH, Sohn MH, Harding MJ, Park JH, Tobiasova Z, Kim DY, Maher SE, Chae WJ, Park SH, Lee CG, Lee SK, Bothwell AL (2010). Cell-permeable Foxp3 protein alleviates autoimmune disease associated with inflammatory bowel disease and allergic airway inflammation. Proc Natl Acad Sci U S A.

[46] Vasconcelos L, Parn K, Langel U (2013). Therapeutic potential of cell-penetrating peptides. Ther Deliv.

[47] Hayes M, Zavazava N (2013). Strategies to generate induced pluripotent stem cells. Methods Mol Biol.

[48] Guo J, Wang H, Hu X (2013). Reprogramming and transdifferentiation shift the landscape of regenerative medicine. DNA Cell Biol.

[49] Fan X, Jin WY, Lu J, Wang J, Wang YT (2014). Rapid and reversible knockdown of endogenous proteins by peptide-directed lysosomal degradation. Nat Neurosci.

[50] Koren E, Apte A, Sawant RR, Grunwald J, Torchilin VP (2011). Cell-penetrating TAT peptide in drug delivery systems: proteolytic stability requirements. Drug Deliv.

[51] Jobin ML, Blanchet M, Henry S, Chaignepain S, Manigand C, Castano S, Lecomte S, Burlina F, Sagan S, Alves ID (2015). The role of tryptophans on the cellular uptake and membrane interaction of arginine-rich cell penetrating peptides. Biochim Biophys Acta.

[52] Tang H, Yin L, Kim KH, Cheng J (2013). Helical Poly(arginine) Mimics with Superior Cell-Penetrating and Molecular Transporting Properties. Chem Sci.

[53] Montrose K, Yang Y, Krissansen GW (2014). The tetrapeptide core of the carrier peptide Xentry is cell-penetrating: novel activatable forms of Xentry. Sci Rep.

[54] Pesce D, Wu Y, Kolbe A, Weil T, Herrmann A (2013). Enhancing cellular uptake of GFP via unfolded supercharged protein tags. Biomaterials.

[55] Farkhani SM, Valizadeh A, Karami H, Mohammadi S, Sohrabi N, Badrzadeh F (2014). Cell penetrating peptides: efficient vectors for delivery of nanoparticles, nanocarriers, therapeutic and diagnostic molecules. Peptides.

[56] Bullenkamp J, Gaken J, Festy F, Chong EZ, Ng T, Tavassoli M (2015). Apoptin interacts with and regulates the activity of protein kinase C beta in cancer cells. Apoptosis.

[57] Backos DS, Brocker CN, Franklin CC (2010). Manipulation of cellular GSH biosynthetic capacity via TAT-mediated protein transduction of wild-type or a dominant-negative mutant of glutamate cysteine ligase alters cell sensitivity to oxidant-induced cytotoxicity. Toxicol Appl Pharmacol.

[58] Gautam A, Chaudhary K, Kumar R, Sharma A, Kapoor P, Tyagi A, Raghava GP, Open source drug discovery c (2013). *In silico* approaches for designing highly effective cell penetrating peptides. J Transl Med.

[59] Kaur H, Garg A, Raghava GP (2007). PEPstr: a de novo method for tertiary structure prediction of small bioactive peptides. Protein Pept Lett.

[60] Willard L, Ranjan A, Zhang H, Monzavi H, Boyko RF, Sykes BD, Wishart DS (2003). VADAR: a web server for quantitative evaluation of protein structure quality. Nucleic Acids Res.

[61] Xu W, Hellerbrand C, Kohler UA, Bugnon P, Kan YW, Werner S, Beyer TA (2008). The Nrf2 transcription factor protects from toxin-induced liver injury and fibrosis. Lab Invest.

[62] Toda K, Kumagai N, Kaneko F, Tsunematsu S, Tsuchimoto K, Saito H, Hibi T (2009). Pentoxifylline prevents pig serum-induced rat liver fibrosis by inhibiting interleukin-6 production. J Gastroenterol Hepatol.

[63] Sundaresan NR, Gupta M, Kim G, Rajamohan SB, Isbatan A, Gupta MP (2009). Sirt3 blocks the cardiac hypertrophic response by augmenting Foxo3a-dependent antioxidant defense mechanisms in mice. J Clin Invest.

