# Supplementary material for: Efficient therapeutic delivery by a novel cell-permeant peptide derived from KDM4A protein for antitumor and antifibrosis
Source: Oncotarget. 2016 Apr 11;7(31):49075–90. doi: 10.18632/oncotarget.8682 (PMC5226491; doi:10.18632/oncotarget.8682)
Supplement: Supplementary file 1 [file oncotarget-07-49075-s001.pdf]

# Efficient therapeutic delivery by a novel cell-permeant peptide derived from KDM4A protein for antitumor and antifibrosis

## Supplementary Materials

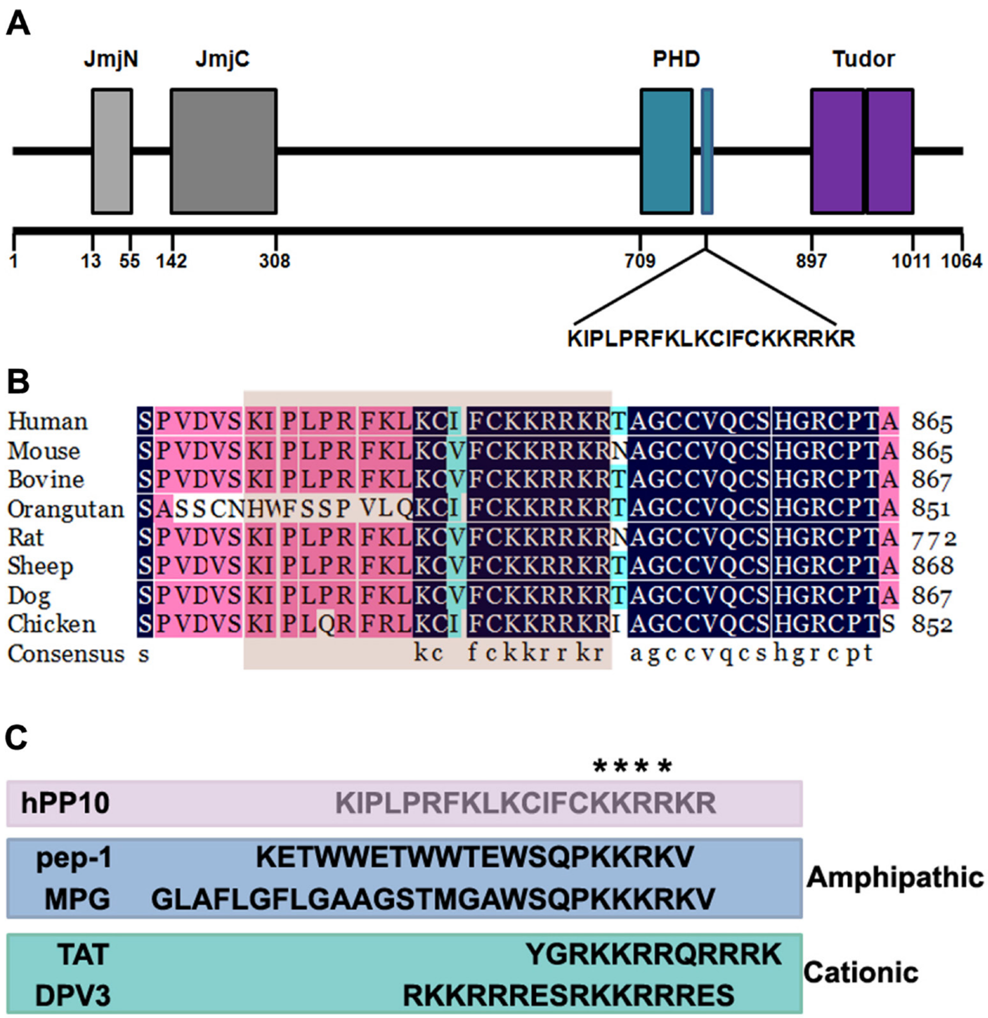


**Supplementary Figure S1: Schematic diagram of JMJD4A protein.** (**A**) Schematic diagram of JMJD4A protein which had various known domains, hPP10 is the fragment of JMJD4A from residue 820 to residue 839. (**B**) JMJD4A sequences are highly conserved among species. The sequences of JMJD4A (also known as KDM4A) from various species were aligned using the DNAman multiple sequence alignment program. The dark blue indicates the conserved region of the JMJD4A-sequence among the various species and the grey box indicates the region of hPP10. (**C**) The sequence alignment of hPP10 and other CPPs shows great conservation of Arg residues.

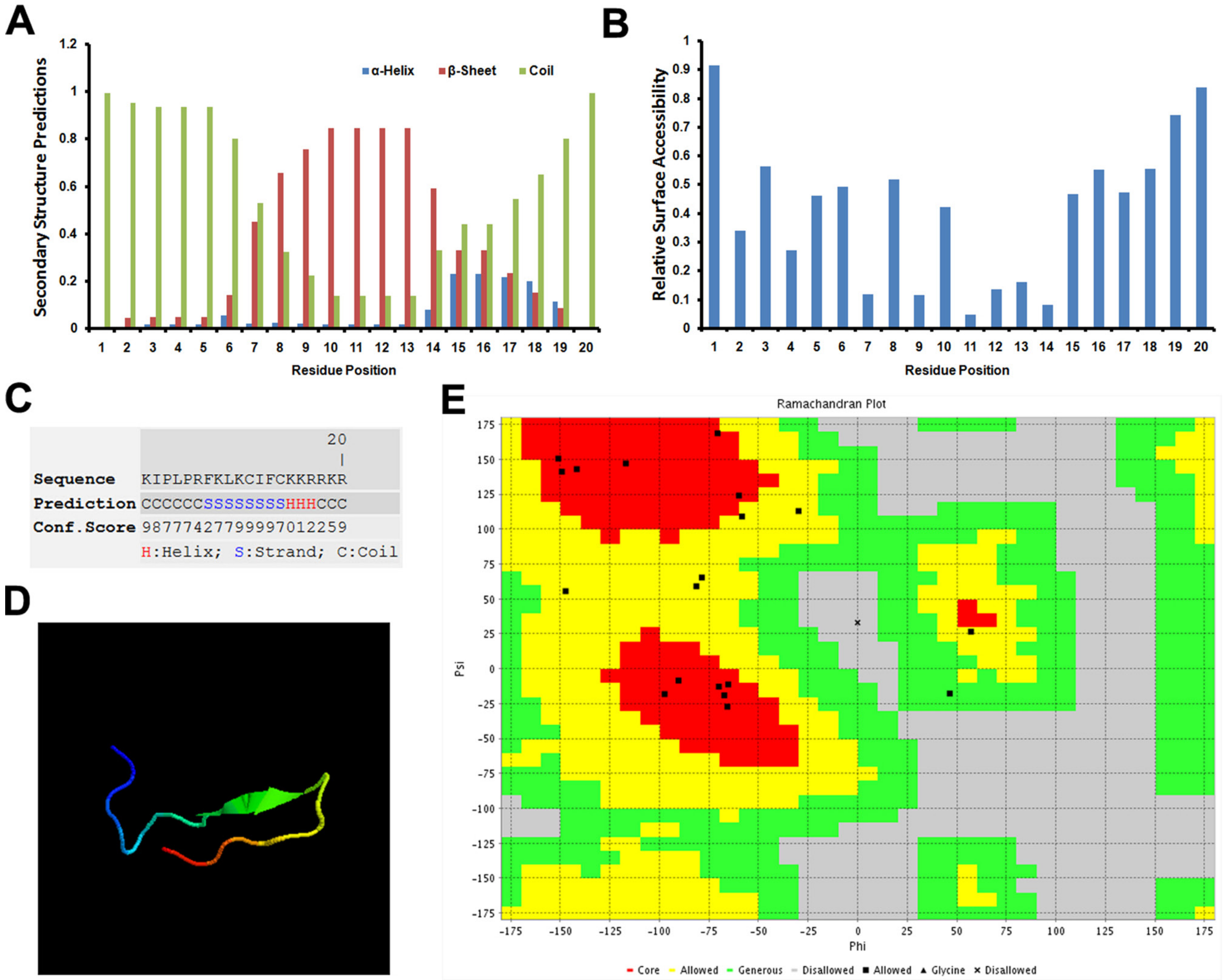


**Supplementary Figure S2: The secondary structure prediction of hPP10.** (**A**) Secondary structure prediction of hPP10 was obtained from the NetSurfP web server (http://www.cbs.dtu.dk/services/NetSurfP/). (**B**) Relative solvent accessibility prediction of hPP10. (**C**) The predicted secondary structure of hPP10. (**D**) The structural model of hPP10 predicted and generated by the I-TASSER server. (**E**) Ramachandran plot analysis of hPP10, all non-glycine and proline residues are shown as filled black squares.

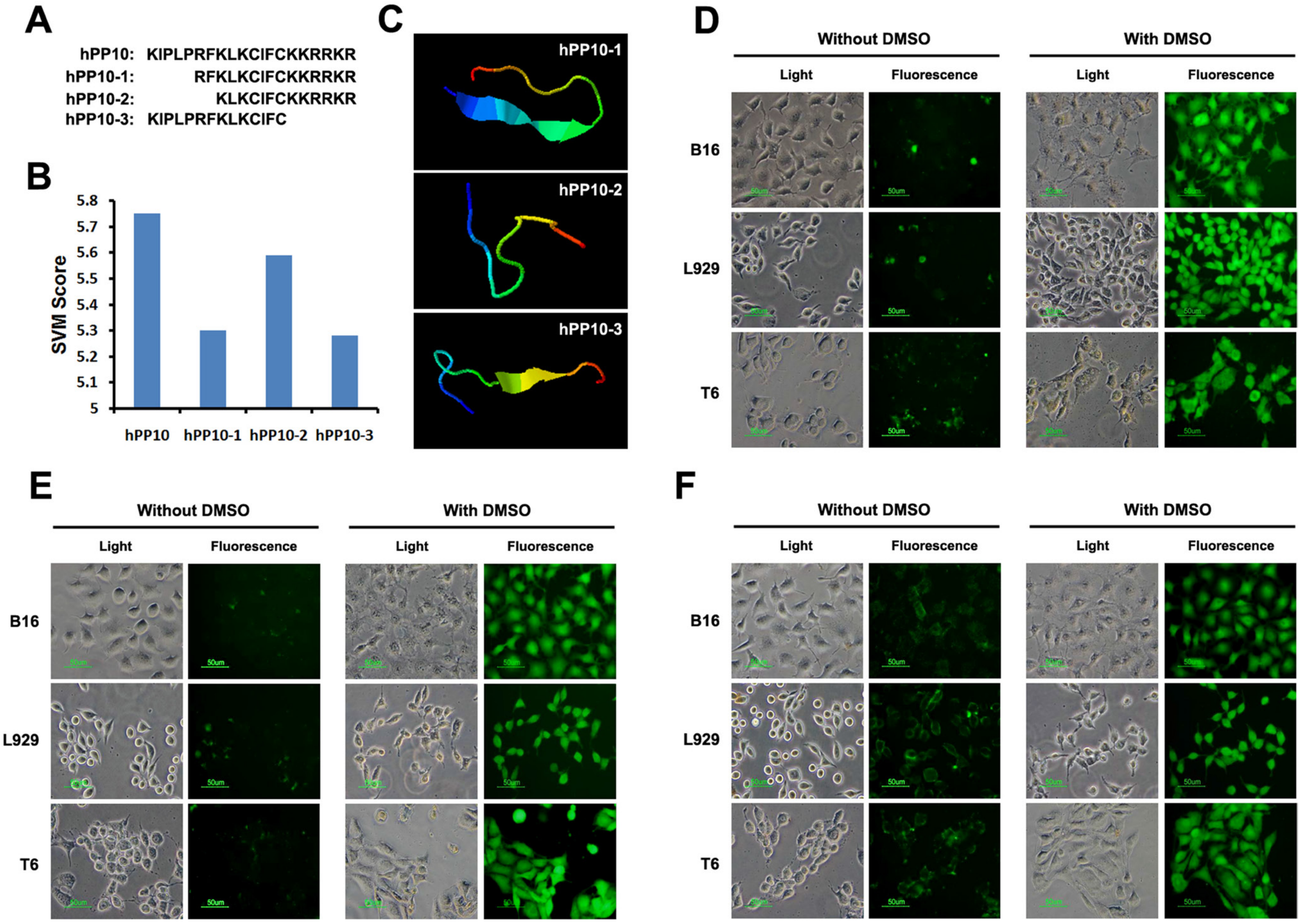


**Supplementary Figure S3: Penetrating efficiency comparsion between truncated hPP10 peptide.** (**A**) Schematic diagram of truncated hPP10 peptide. (**B**) SVM score of truncated hPP10 by CellPPD server. (**C**) The structural model of truncated hPP10. (**D**) Penetration efficiency of hPP10-1 in different cell lines with or without DMSO. (**E**) Penetrating efficiency of hPP10-2 in different cell lines with or without DMSO. (**F**) Penetration efficiency of hPP10-3 in different cells with or without DMSO.

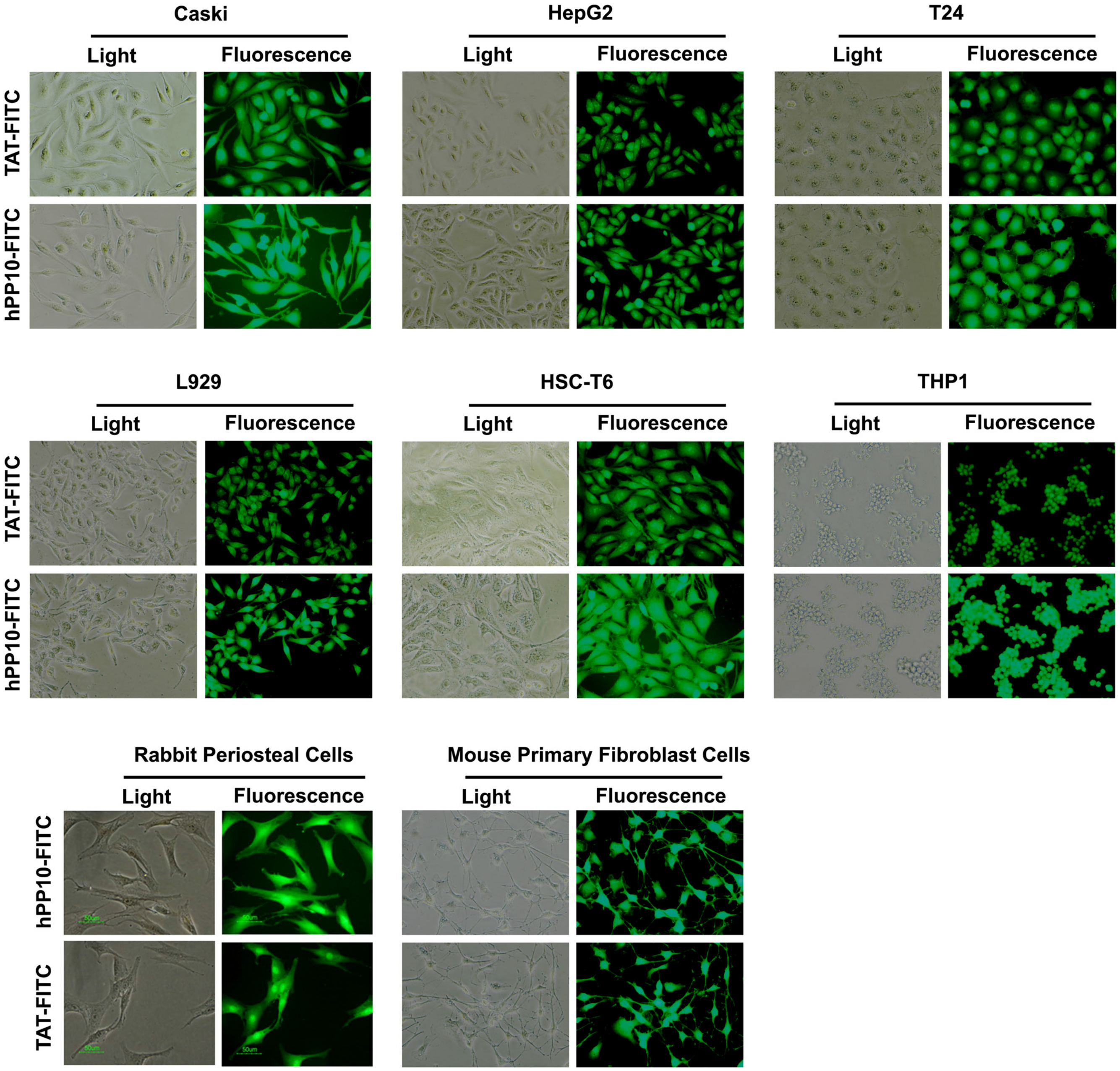


**Supplementary Figure S4: Penetration of hPP10-FITC in different type of cells, including cancer cell lines, normal cell lines as well as primary cultured cells.**

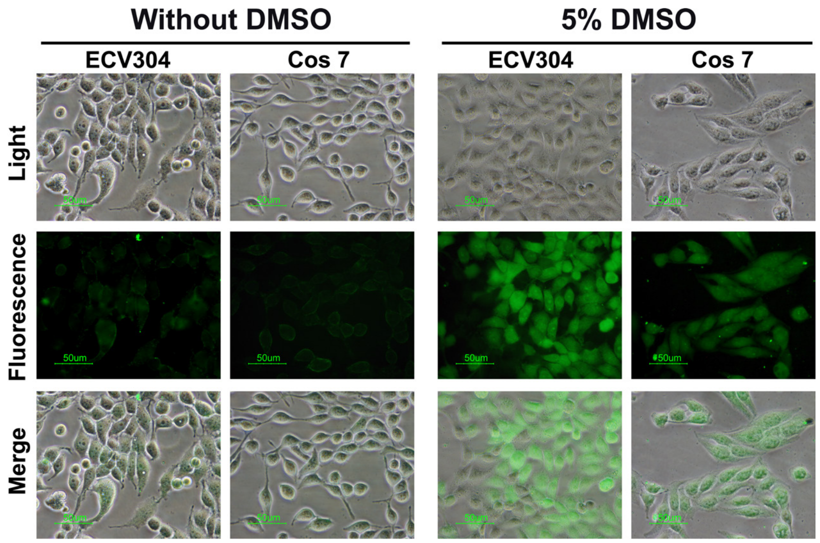


**Supplementary Figure S5: Penetration of hPP10-GFP in cultured ECV304 and Cos 7 cells.**

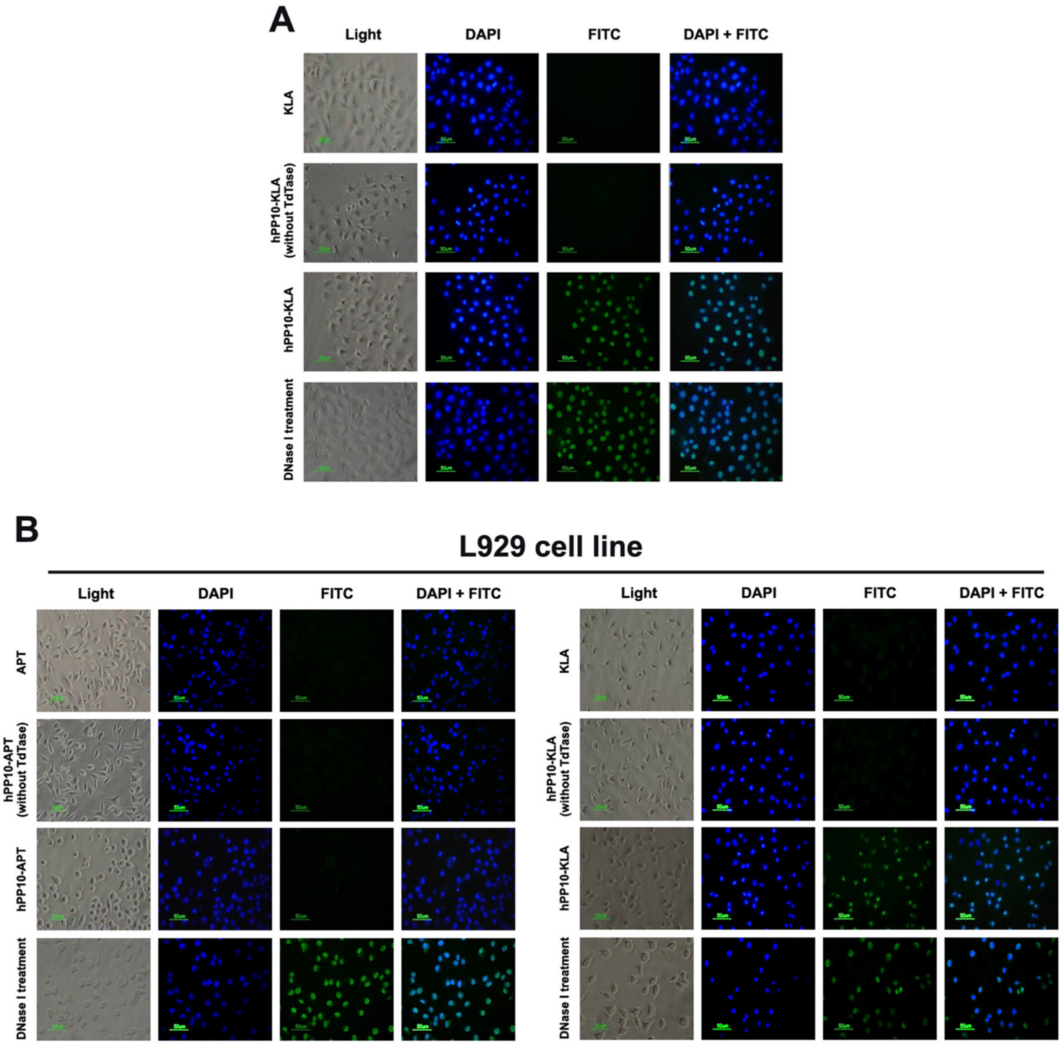


**Supplementary Figure S6: TUNEL assay after hPP10-Apoptin or hPP10-KLA treatment.** (**A**) B16 cells apoptosis detected after hPP10-KLA treatment. (**B**) L929 cells treated by hPP10-Apoptin cannot induce L929 cells apoptosis, while hPP10-KLA can induce L929 cells apoptosis.

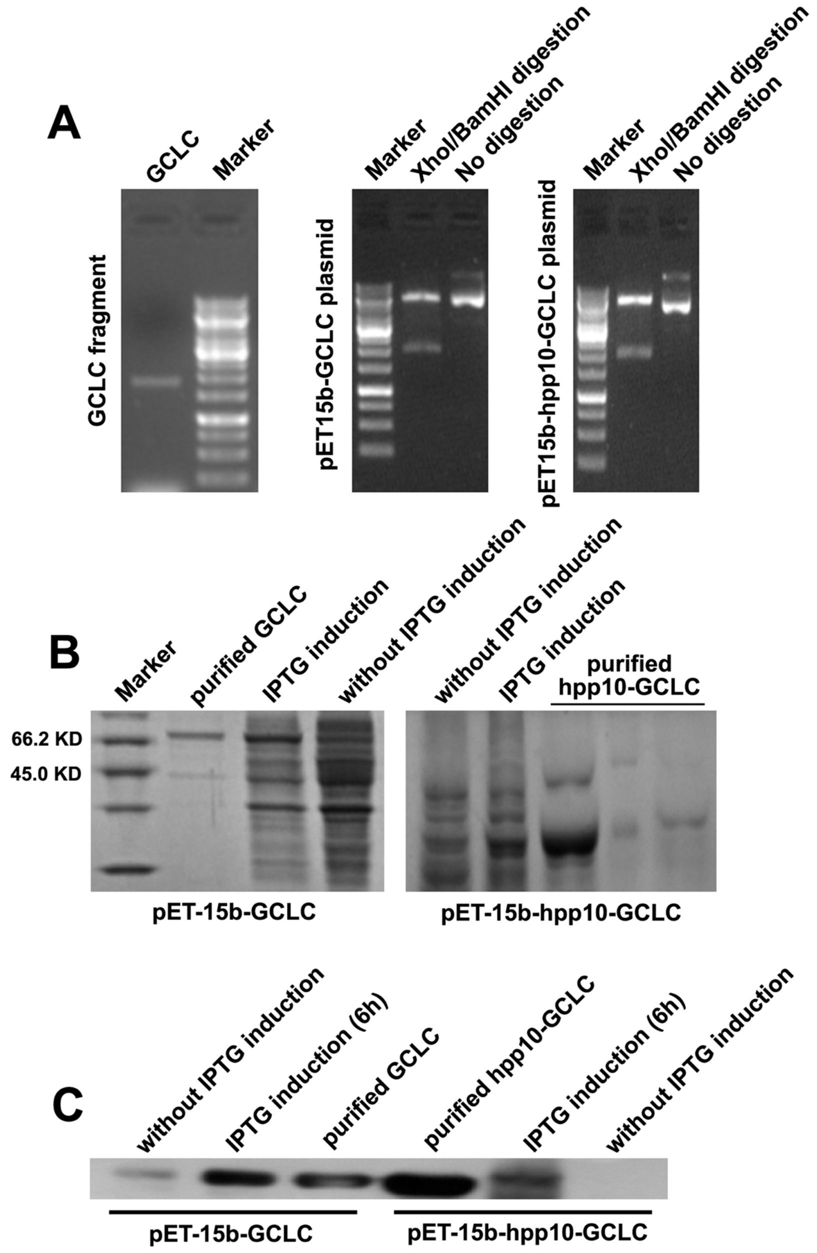


**Supplementary Figure S7: Recombinant plasmid construction, fusion protein expression and its characterization.** (**A**) Recombinant plasmid construction. (**B**) Fusion protein of GCLC or hPP10-GCLC expression. (**C**) Recombinant fusion protein characterization by Western blotting.

**Supplementary Table S1: Name, sequence, charge, and CellPPD prediction scores of peptides identified from SwissProt database**

| Designation | Sequence | SVM Score | Protein Name | SwissProt ID | Region (aa) | Arg | Charge |
|---|---|---|---|---|---|---|---|
| hPP1 | RRKHRRRRRRSRTFSRSSSM | 5.6 | CLK2 | P49760 | 115–134 | 10 | 11 |
| hPP2 | SVSIALSWRRRRRRKKRRSS | 5.5 | MYL4 | I3L532 | 63–82 | 8 | 10 |
| **hPP3** | **KPKRKRRKKKGHGWSRMGTR** | **5.22** | **SP140L** | **Q9H930** | **207–226** | **5** | **11** |
| hPP4 | RLLRCQCRRRRRRRPLWRVY | 5.53 | ADRA1D | P25100 | 415–434 | 10 | 10 |
| hPP5 | KQPRVQRPRPRRRRRRRTNG | 5.71 | BAHD1 | Q8TBE0 | 571–590 | 10 | 11 |
| hPP6 | MMMRRTKRRRRKRRRMTMMT | 5.61 | ZBTB7C | L8E8J6 | 12–30 | 9 | 11 |
| hPP7 | RRRRKRVNTKRSSRAFRAHL | 5.42 | DRD2 | P14416 | 217–236 | 9 | 10 |
| hPP8 | SALRFHKRNTITKRRKKRNR | 5.44 | PHIP | Q8WWQ0 | 1427–1446 | 6 | 10 |
| hPP9 | RQQRRRKRRRSTRRSTRSES | 5.6 | ZCCHC17 | A0A087WYF0 | 134–153 | 10 | 11 |
| hPP10 | **KIPLPRFKLKCIFCKKRRKR** | **5.75** | **KDM4A** | **O75164** | **820–839** | **5** | **10** |
| hPP11 | RGRASKGTGKRRKKRPSRSQ | 5.33 | INF2 | Q27J81 | 1211–1230 | 6 | 10 |
| hPP12 | QVPKKPKRKKRRRRNVNCLK | 5.27 | ZNF653 | Q96CK0 | 105–124 | 5 | 11 |
| hPP13 | KLNVFSRVKLFGSKKRRRRR | 5.55 | FGF13 | Q92913 | 42–61 | 6 | 10 |
| hPP14 | AGAQASSKHQKRKKRRKRQK | 5.34 | NPAS3 | Q8IXF0 | 589–608 | 4 | 10 |
| hPP15 | PPRIRPQKKRKRRVYKVRTS | 5.29 | ZDHHC1 | I3L202 | 231–250 | 6 | 10 |
| hPP16 | KQNKKKKRKKRKSPKSSLLN | 5.32 | PCDH9 | Q9HC56 | 864–883 | 2 | 11 |
| hPP17 | VSRAGSSLQKKRKKRKHLRK | 5.35 | DHX16 | Q6PKC8 | 111–130 | 4 | 10 |
| hPP18 | GKNVPSVWRSARRKRKKKHR | 5.44 | TAF1 | P21675 | 248–267 | 5 | 10 |
| hPP19 | QRFRKKRRRSQRRPRLRQFS | 5.31 | TRABD2A | Q86V40 | 417–436 | 9 | 11 |
| hPP20 | MMMMKMMRRRKKRRRKSLSK | 5.38 | NCL | L8EAF1 | 21–40 | 6 | 11 |
| hPP21 | TRKKRRRSSSYSPSPVKKKK | 5.52 | SRRM4 | A7MD48 | 117–136 | 4 | 10 |
| hPP22 | KHKRKKRRKQNQNQHRSRHR | 5.48 | LINC00467 | Q9BRT7 | 21–40 | 6 | 11 |
| hPP23 | RKKRRKFWKAKNLQHIPLKK | 5.55 | KLB | Q49AQ7 | 280–299 | 3 | 10 |
| hPP24 | RRKKRKKISGPNPLSCLKKK | 5.56 | UTP23 | Q9BRU9 | 192–211 | 3 | 10 |
| hPP25 | QSSASRLMRRHKRRRRKQKV | 5.49 | DVL3 | Q92997 | 207–226 | 7 | 10 |
| hPP26 | RKMSKSALNQTKKRKKRRHR | 5.44 | VSX2 | P58304 | 133–152 | 5 | 11 |
| hPP27 | VAPCKPKKKRRRKRGRFHPG | 5.35 | ZNF804A | Q7Z570 | 803–822 | 5 | 10 |
| hPP28 | AGCLLNRRRAARRRRKRLRQ | 5.55 | IGSF9 | Q9P2J2 | 740–759 | 9 | 10 |
| hPP29 | ALKKASKRRKRRKNVFVHKK | 5.41 | SFMBT1 | Q9UHJ3 | 660–679 | 4 | 11 |
| hPP30 | AAHWLMRRRRRKQRKKKAWI | 5.49 | TP53I13 | Q8NBR0 | 125–144 | 6 | 10 |
| hPP31 | AGTASTGRRKRRRRRKPKQK | 5.64 | LPIN3 | Q9BQK8 | 134–153 | 7 | 11 |
| hPP32 | RCRSQPCVLSGKRSRRKRRR | 5.45 | FAM53A | Q6NSI3 | 257–276 | 8 | 10 |
| hPP33 | **FLLDRKKTDKLKKKKKRKRR** | **5.66** | **PHF13** | **Q86YI8** | **106–125** | **4** | **13** |

**Supplementary Table S2: CPP prediction and physicochemical properties of hPP10 by CellPPD server, hPP10 and its internal fragments were evaluvated for SVM score, prediction, hydrophobicity, stearic hindrance, side bulk, hydropathicity, amphipathicity, hydrophilicity, net hydrogen, charge, pI and molecular weight.**
