# Supplementary material for: Efficient therapeutic delivery by a novel cell-permeant peptide derived from KDM4A protein for antitumor and antifibrosis
Source: Oncotarget. 2016 Apr 11;7(31):49075–90. doi: 10.18632/oncotarget.8682 (PMC5226491; doi:10.18632/oncotarget.8682)
Supplement: Supplementary file 2 [file oncotarget-07-49075-s002.docx]

**Supplementary information.**

**Supplementary Table 2.** CPP prediction and physicochemical properties of hPP10 by CellPPD server, hPP10 and its internal fragments were evaluvated for SVM score, preduction, hydrophobicity, stearic hindrance, side bulk, hydropathicity, amphipathicity, hydrophilicity, net hydrogen, charge, pI and molecular weight.
